# Nickel Thiazoledithiolenes: π-Extended Fused-Ring Metal Dithiolenes as Highly Delocalized π-Electron Systems with Stabilized Frontier Orbitals

**DOI:** 10.3390/molecules30193998

**Published:** 2025-10-06

**Authors:** Eric J. Uzelac, Juan Sánchez-Rincón, M. Carmen Ruiz Delgado, Seth C. Rasmussen

**Affiliations:** 1 Department of Chemistry and Biochemistry, North Dakota State University, NDSU Dept. 2508, P.O. Box 6050, Fargo, ND 58108-6050, USA; 2 Department of Physical Chemistry, University of Málaga, Campus de Teatinos s/n, 29071 Málaga, Spain; 3 Instituto Universitario de Materiales y Nanotecnología, IMANA, University of Málaga, 29071 Málaga, Spain

**Keywords:** metal dithiolenes, nickel thiophenedithiolenes, nickel thiazoledithiolenes, non-innocent ligands, structure–function relationships, conjugated materials

## Abstract

Building off previous work on π-extended nickel thiophenedithiolenes, a series of thiazole-fused nickel dithiolene complexes have been prepared via similar synthetic methods, thus allowing for the addition of aryl groups to the terminal α-position of the fused thiazoledithiolene unit. In addition to π-extended complexes incorporating thiophene, phenyl, and furan end-groups, the methyl-terminated species has also been prepared as a representative of the simple nickel thiazoledithiolene core. The optical, electronic, and structural properties of these complexes have been characterized, and comparisons to the analogous nickel thiophenedithiolenes show that the replacement of thiophene by thiazole stabilizes the frontier orbitals of the thiazole-based complexes, while preserving the planar geometry, electronic delocalization, and low-energy NIR absorption of the previous nickel thiophenedithiolene species.

## 1. Introduction

**Figure 1:**
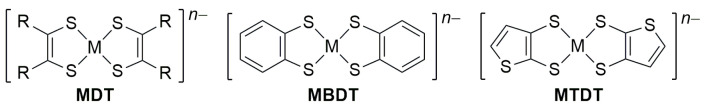
Examples of four-coordinate metal dithiolenes.

Metal dithiolenes, complexes of bidentate thiolate ligands conjugated via a carbon–carbon double bond (MDT, Figure 1), date back to initial reports in 1962–1963 [1] and have grown to become species of significant focus due to their rich electronic and magnetic properties. These features, coupled with their bulk solid-state packing, have led to materials exhibiting notable conductivity [2,3,4,5,6,7,8,9], superconductivity [2,3], ferromagnetism [2,4,7,9], and non-linear optical response [2,8,10]. Because of this, metal dithiolenes have been widely studied as building blocks for crystalline molecular materials, as well as for potential coordination polymers and metal-containing conjugated polymers [6,7,9,11,12]. Finally, with the proper terminal functional groups, metal dithiolenes can also exhibit liquid crystalline properties, thus allowing for field-induced absorbance switching [13].

The desired electronic properties and solid-state packing of these complexes can then be tuned with the choice of central metal center or through the functional groups included on the dithiolene moiety. In addition, an alternate approach to tuning the material properties has been through the fusion of aromatic rings to the dithiolene core. Common examples of such fused-ring metal dithiolenes have included metal benzenedithiolenes (MBDT, Figure 1) [11,14,15,16,17,18] and metal thiophenedithiolenes (MTDT) [6,7,9,12,19,20], with the added ring fusion increasing molecule planarity and enhancing orbital overlap, thus maximizing electron delocalization into the organic ligands.

**Figure 2:**
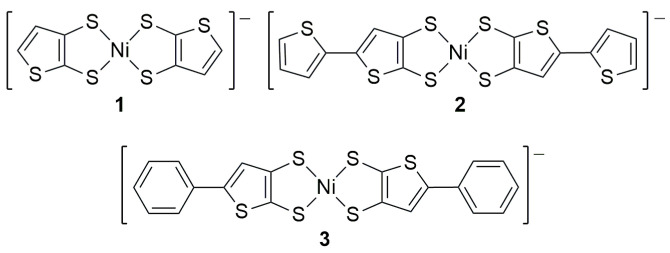
Nickel thiophenedithiolene and its π-extended analogues.

With the goal of producing new hybrid materials that combine the characteristics of metal dithiolenes and oligothiophenes [21,22], the Rasmussen group developed methods for the synthesis of π-extended analogues of the known nickel thiophenedithiolene core (**1**, Figure 2) [9,23,24]. This included the thiophene-extended analogue **2** and its derivatives, as well as the phenyl-extended analogue **3**, all of which exhibited unprecedented electronic delocalization, with low-energy NIR transitions in the range of 1076–1160 nm [23,24]. In addition, these π-extended nickel thiophenedithiolenes retained the characteristics of typical metal dithiolenes including rich electrochemistry, close contacts in the solid state, and magnetic or semiconducting properties, thus providing significant promise for their application to optoelectronic devices.

In order to investigate the ability to tune the electronic properties of this new scaffold, efforts then turned to the development of π-extended metal thiazoledithiolenes, in which the fused thiophene of the nickel thiophenedithiolene core is replaced with the more electron-deficient thiazole. The replacement of thiophene with thiazole has previously been shown to be a quite successful approach for stabilizing the frontier orbitals of π-conjugated units, while having little-to-no effect on the corresponding structural properties [25,26]. As such, this seemed like a logical way to further extend this class of π-extended fused-ring metal dithiolenes.

**Figure 3:**
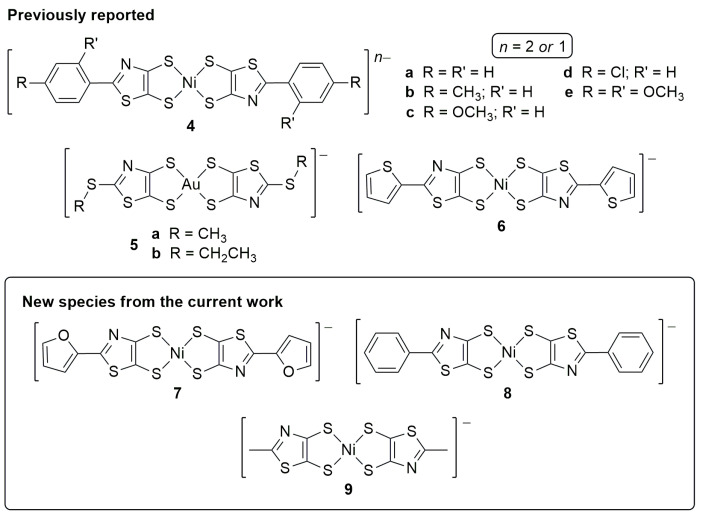
Known nickel thiazoledithiolene derivatives.

To date, there have been very few reports of such metal thiazoledithiolenes. In a brief 1988 communication [27], Kibbel and coworkers reported the preparation of a series of phenyl-extended nickel thiazoledithiolenes (**4a**–**e**, Figure 3). However, very little synthetic details were provided, and the reported product characterization was extremely limited. The authors also stated that the analogous palladium species were produced by similar methods, but no details or evidence was provided to support such a claim. Lorcy and coworkers then reported additional routes to the gold thiazoledithiolenes **5a**–**b** beginning in 2015 [28,29]. Unfortunately, the synthetic methods developed for these species were specifically limited to 2-alkylthio derivatives and could not be more broadly applied. Finally, the Rasmussen group applied the methods developed for the previous π-extended nickel thiophenedithiolenes to generate the thiophene-extended nickel thiazoledithiolene **6** in 2017 [30]. As hoped, compound **6** exhibited very similar properties to the previous thiophenedithiolene analogue **2**, but with stabilization of the frontier orbitals by ca. 0.2 eV. No further examples of metal thiazoledithiolenes have been reported since.

In order to continue the study of these metal thiazoledithiolenes, the family of π-extended nickel thiazoledithiolenes has now been expanded to include both the furan and phenyl analogues of nickel thiazoledithiolene **6**. In addition, the methyl-capped species **9** has been prepared as the closest known analogue to the simple nickel thiazoledithiolene core. This will allow quantification of the effect of the π-extension in the species **6**–**8**, as well as direct comparison to the analogous thiophene-based core **1**. Of course, it should be pointed out that technically the new species **8** should be identical to the previous monoanion **4a** reported by Kibbel and coworkers. However, compound **8** has been specifically given a different compound number here to better clarify that it is prepared by different methods. Furthermore, as part of the presented study, these two species will be directly compared to determine whether or not they are truly the same chemical species.

## 2. Results and Discussion

### 2.1. Synthesis

**Scheme 1:**
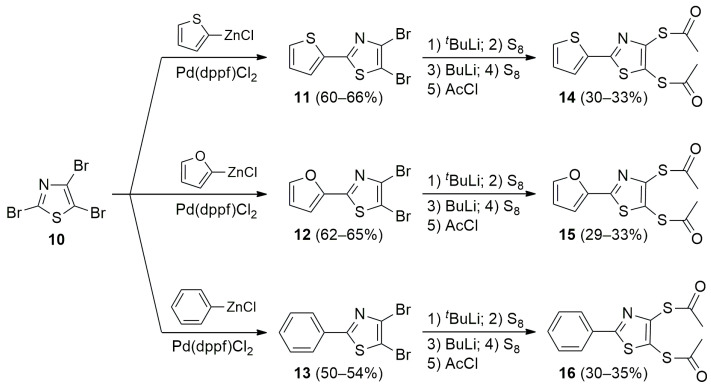
Synthesis of acetyl-protected thiazoledithiolate ligands.

The π-extended nickel thiazoledithiolenes **6**–**8** were prepared in a similar manner to the previously reported nickel thiophenedithiolene analogues **2** and **3** [23,24]. As given in Scheme 1, regioselective cross-coupling methods originally developed for 2,3,5-tribromothiophene [31] were first applied to 2,4,5-tribromothiazole (**10**) [32] to generate the desired 2-aryl-4,5-dibromothiazole intermediates **11**–**13** in reasonable yields (ca. 50–66%). The intermediates **11**–**13** were then converted to the acetyl-protected thiazoledithiolate ligands **14**–**16** via a one-pot, sequential process as shown in Scheme 1. The details of the specified sequential addition steps here are critical to limit side reactions, the most critical of which is the need to inhibit the halogen dance reaction that can lead to unwanted rearrangements of lithiated thiophenes and their heterocyclic analogues [33]. Thus, the use of *tert*-butyllithium (*^t^*BuLi) is necessary in the first step, as the resulting *tert*-butylbromide formed is relatively inert to nucleophilic substitution by the sulfide formed in the second step. In contrast, it was found during the development of these methods that the use of simple butyllithium (BuLi) in the first step resulted in the formation of S-alkylated byproducts. In order to inhibit the halogen dance reaction, the addition of the third step is inverted, such that the reaction mixture following the second step was then added to a fresh solution of excess BuLi, a technique known to suppress the halogen dance reaction [33]. Although the resulting yields of **14**–**16** are somewhat limited, this is in good agreement with the previously reported thiophene species [23,24] and illustrates that the electronic differences between thiophene and thiazole have little effect on the synthetic methods employed.

**Scheme 2:**
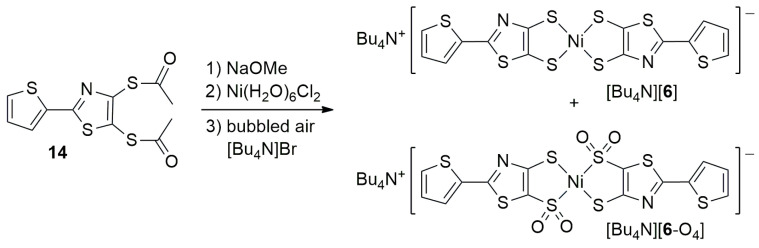
Initial synthesis of thiophene-extended nickel thiazoledithiolate **6**.

As our group had previously reported [30], initial attempts to synthesize the thiophene-extended nickel thiazoledithiolene **6** via the same methods as previously used for the thiophenedithiolene analogue **4** were found to give a mixture of products. As detailed in Scheme 2, this included the tetrabutylammonium [Bu_4_N] salt of the desired compound **6** and a second product in which two of the metal-coordinated sulfur atoms had been doubly oxidized to sufonyls (**6**-O_4_), with both products [Bu_4_N][**6**] and [Bu_4_N][**6**-O_4_] confirmed by X-ray crystallography [30]. Although sulfur-oxidized dithiolene complexes of this type have been previously reported [2,34,35,36,37,38], their production generally requires the application of radical oxidants such as peroxides. Such sulfur oxidation via O_2_ is known, but has required either excessively strong alkaline conditions [37] or strong electron-withdrawing groups [38]. Still, the fact that such sulfur oxidation occurs via the simple bubbling of air for **6**, but is not observed for the analogous nickel thiophenedithiolenes, does illustrate the extent that the ligand electronics have been modified by the replacement of thiophene with thiazole.

**Scheme 3:**
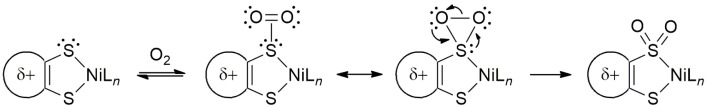
Proposed mechanism of sulfur oxidation via O_2_.

It has been proposed that the direct reaction of dithiolenes with molecular oxygen could occur via the formation of oxygen adducts with one of the metal-coordinated sulfurs [37]. As shown in Scheme 3, such an adduct would involve the O_2_ molecule acting as a σ donor via overlap of its π bond and an empty sulfur *d* orbital. Such an interaction would be more favorable in cases in which electron density is pulled away from the sulfur, which is consistent with both the observed reactivity of the previously reported nickel benzothiadiazole-fused dithiolene [38] and the nickel thiazoledithiolene here. Backbonding from one of the filled sulfur orbitals into the O_2_ π* orbital would then contribute to a resonance structure consisting of the three-membered ring given in Scheme 3. Finally, ring opening would result in the formal sulfonyl product. As the formation of this sulfonyl would further pull electron density from the metal complex, this would then make it more favorable for a second sulfur to form another adduct with oxygen, ultimately leading to the observed product **6**-O_4_.

One aspect that separates the formation of **6**-O_4_ from previous sulfur-oxidized dithiolene complexes is that reaction between O_2_ and **6** results in both oxidation of two of the dithiolene sulfurs and oxidation of the metal complex from the dianion to the monoanion. In comparison, previous examples resulted in sulfur oxidation without change in the overall complex charge. While it is unclear the order of oxidation processes, oxidation of **6** from the initial dianion to the monoanion would remove electron density and thus favor formation of the initial oxygen adduct proposed in Scheme 3.

**Scheme 4:**
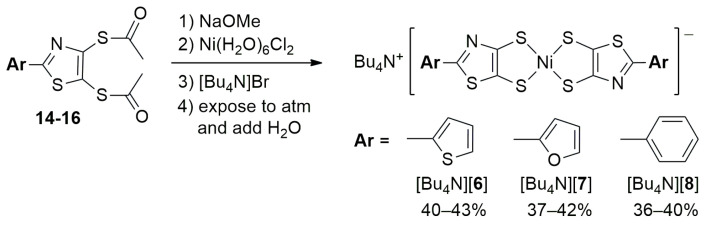
Optimized synthesis of π-extended nickel thiazoledithiolenes.

As previously reported [30], it was found that sulfur oxidation could be inhibited by eliminating the air bubbling and simply opening the reaction mixture to ambient atmosphere, followed by the addition of non-deoxygenated water to prompt mild oxidation and precipitate the monoanion salt [Bu_4_N][**6**]. As these modified methods allowed the selective production of the desired π-extended nickel thiazoledithiolene **6**, this was then applied to the production of the additional species **7** and **8** as shown in Scheme 4. In this way, the π-extended nickel thiazoledithiolenes **6**–**8** were isolated as dark green Bu_4_N salts in yields of 36–43%.

In order to allow for the quantification of the effects of the pendent aryl groups, attempts were then made to synthesize the core nickel thiazoledithiolene complex (NiTzDT), which would also allow direct comparison to the thiophenedithiolene analogue **1**. Initial attempts began with subjecting 4,5-dibromothiazole (**17**) [32] to our standard conditions used for converting dibromothiazoles to their corresponding acetyl-protected thiazoledithiolate ligands (Scheme 1). Such efforts were unsuccessful, however, which was attributed to the more acidic nature of the hydrogen at the 2-position of the thiazole [39].

**Scheme 5:**
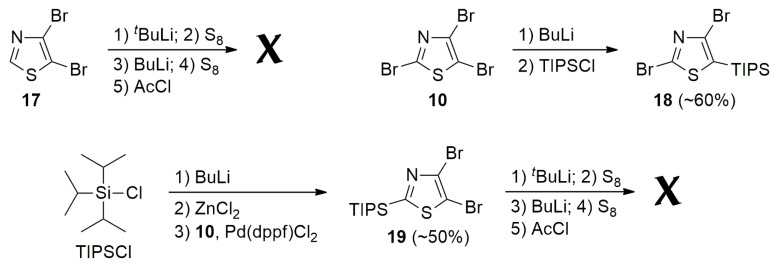
Attempted synthesis of unfunctionalized and TIPs-protected thiazoledithiolate ligands.

Efforts then moved to approaches to block the 2-position to prevent deprotonation (Scheme 5). As previous studies with thiazole species had employed the triisopropylsilyl (TIPS) group for this, 2,4,5-tribromothiazole (**10**) was debrominated with BuLi in the presence of TIPSCl. The hope here was that substitution of the lithiated thiazole would thus take place before any potential rearrangement via the halogen dance [33]. Unfortunately, this was not the case, with the isolated product found to be 2,4-dibromo-5-triisopropylsilylthiazole (**18**), rather than the desired 4,5-dibromo-2-triisopropylsilylthiazole (**19**). As such, it was determined that the rate of the halogen dance exchange surpassed that of the reaction with TIPSCl.

An alternate path to **19** was then attempted based on the previously successful Negishi cross-coupling pathway given in Scheme 1. Thus, TIPSCl was treated with BuLi and reacted with ZnCl_2_ to produce the intermediate TIPSZnCl. Negishi cross-coupling between TIPSZnCl and **10** using the sterically bulky Pd(dppf)Cl_2_ then successfully led to the desired species **19** in approximately 50% yield (Scheme 5). Unfortunately, conversion of **10** to the desired acetyl-protected thiazoledithiolate was again unsuccessful. Analysis by ^1^H NMR revealed a complex mixture of thiazole species that included signals corresponding to the naked 2-position (ca. 8.5–9.0 ppm), thus indicating cleavage of the TIPS protecting group.

**Scheme 6:**
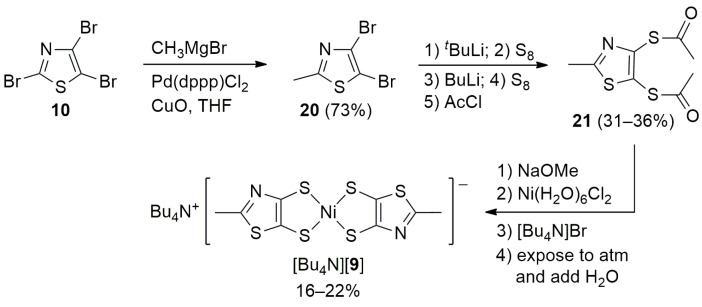
Synthesis of the core nickel thiazoledithiolene complex **9**.

As the application of a protecting group was unsuccessful, it was decided to move to the addition of a full blocking moiety at the 2-position of the thiazole. In order to minimize any structural effects, the smallest possible option would be a simple methyl group. Furthermore, the use of such alkyl groups as terminal blocking units in oligothiophenes are known to have only slight effects on the optical and electronic properties, with each terminal alkyl typically resulting in a 5–10 nm red shift in the oligomer absorption [40]. As given in Scheme 6, Kumada cross-coupling between **10** and CH_3_MgBr successfully gave the desired 4,5-dibromo-2-methylthiazole (**20**). Originally isolated in 53% yield, it was found that the addition of CuO aided the reaction [41], increasing the yield to 73%. The acetyl-protected 2-metylthiazoledithiolate (**21**) was then successfully generated in yields of 31–36% by subjecting **20** to the standard conditions previously developed. Finally, **21** was deprotected with NaOMe and the core nickel thiazoleditholene **9** produced as its Bu_4_N salt via the methods previously applied to the analogous aryl-extended complexes **6**–**8**.

### 2.2. X-Ray Crystallography and Structural Analysis

**Figure 4:**
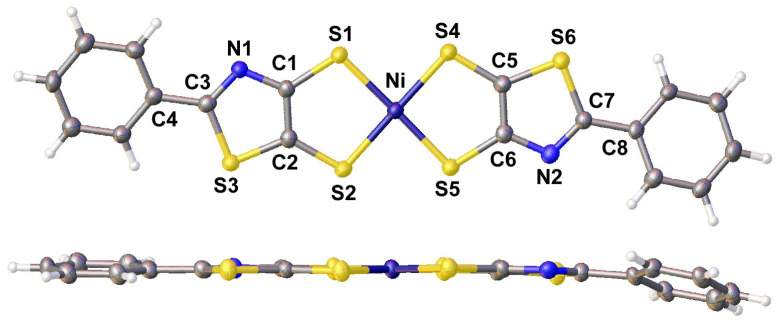
Face and edge ellipsoid plots of **8** at the 50% probability level.

As with the previous aryl-extended nickel thiophenedithiolenes [23,24], the thiazole analogues are amenable to crystallization, allowing the growth of single crystals and structural determinations via X-ray crystallography. The crystal structures have been determined for both of the aryl-extended nickel thiazoledithiolene complexes **6** and **8**, as well as the sulfur-oxidized derivative **6**-O_4_. The Bu_4_N salts of both **6** and **6**-O_4_ were previously reported in 2017 [30], with the additional structure of the salts [Bu_4_N][**8**] reported in this current broader study. Ellipsoid plots of complex **8** are shown in Figure 4, and selected bond distances for the nickel thiazoledithiolene cores of **6** and **8** are given in Table 1, along with thiazole [42] and the nickel thiophenedithiolene analogues **2** and **3** [23,24] for comparison.

As with the previously reported nickel thiazoledithiolene **6** [30], the phenyl-extended analogue **8** exhibits a structure in which the two thiazoledithiolene ligands adopt a *trans* configuration. This *trans* geometry is the most favorable due to its lower energy and is also the most common form observed for the analogous thiophenedithiolenes [7,23,24]. The Ni-S bond lengths for both **6** and **8** range from 2.166 to 2.176 Å, which agree well with metal-sulfur bonds of the thiophenedithiolene analogues **2** and **3**. The fused thiazole rings of both metal complexes also exhibit bond lengths that agree fairly well with those determined for the isolated parent thiazole [42], showing a maximum deviation of ca. 0.03 Å for both **6** and **8**. The slightly increased asymmetry observed within the thiazole ring of the metal complexes is fairly characteristic of fused five-membered heterocycles.

As shown in Figure 4, the nickel thiazoledithiolene **8** is not completely planar, largely due to twists of about 5.3–8.9° around the C3-C4/C7-C8 bonds between the pendent phenyl groups and the nickel thiazoledithiolene core. Similar deviations from planarity are also observed for **6**, with slightly larger twists of 12.7–14.0° between the core and terminal thiophene units [30]. Limiting the analysis to just the planarity of the central nickel thiazoledithiolene core, a minor tetrahedral distortion of the nickel coordination geometry can be seen that results in a small twist along the long axis of the complex of ca. 2.5° and 5° for **8** and **6,** respectively**.** Such small tetrahedral distortions are quite typical of square planar metal dithiolenes [2]. In comparison, the reported structures of the nickel thiophenedithiolene analogues **2** and **3** are much more planar [23,24]. However, it is believed that this is largely due to the fact that these were crystallized as salts of the planar aromatic *N*-methylpyridinium (NMP), rather than due to any particular structural differences resulting from the replacement of thiophene by thiazole. The inclusion of the NMP cation allowed effective π-π stacking between the cation and metal complexes [23,24], thus favoring planar stacked structures.

Comparing the interannular bonds between the nickel thiazoledithiolene core and terminal aryl rings (i.e., C3-C4 and C7-C8), the phenyl-extended complex **8** exhibits an average length of 1.47 Å, slightly longer than the value of 1.44 Å found for the thiophene-extended complex **6**. This effect of the terminal aromatic ring is nearly identical to that seen in the nickel thiophenedithiolene analogues, with average values of 1.51 Å and 1.47 Å for the phenyl- and thiophene-extended species **3** and **2**, respectively [23,24]. This observed effect in both series is also consistent with the view that the greater aromaticity of benzene results in greater electron confinement and thus reduced conjugation [43] or electron delocalization between the external phenyl unit and central metal dithiolene core.

**Figure 5:**
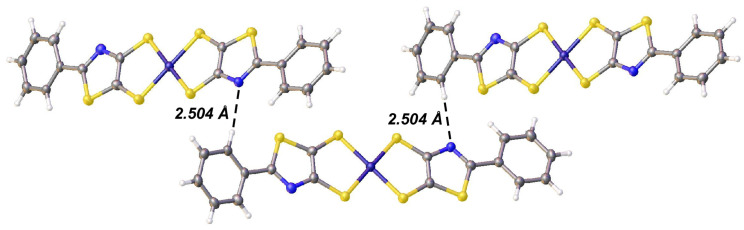
Packing of **8** illustrating intermolecular close contacts.

While the thiophene-extended nickel thiazoledithiolene **6** [30] and the previous π-extended nickel thiophenedithiolenes **2** and **3** [23,24] all exhibit S···S and C–H···S complimentary close contacts between dithiolene neighbors, the phenyl-extended nickel thiazoledithiolene **8** instead only exhibits C–H···N hydrogen bonding as illustrated in Figure 5. Here, the hydrogen bonding is between the C–H at the 2-position of the phenyl group of one complex and the thiazole nitrogen at the opposite end of a neighboring complex, with each complex acting both as a hydrogen bond donor at one end and a hydrogen bond acceptor at the other. The orientation of the complexes making up each hydrogen bond interaction is canted (ca. 46°), resulting in an overall distorted lattice-work type assembly. The hydrogen bonds in all cases exhibit an estimated H···N distance of 2.50 Å (less than the sum of the van der Waals radii of 2.75 Å [44]), with a corresponding C–H···N angle of 169.5°.

### 2.3. Electrochemistry

**Figure 6:**
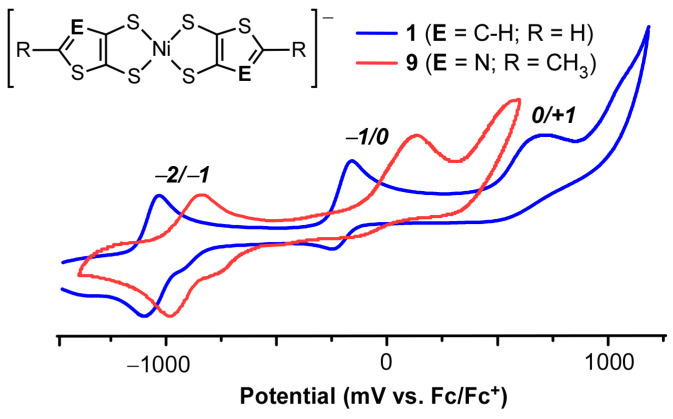
Cyclic voltammograms of the parent nickel thiophenedithiolene **1** and nickel thiazoledithiolene **9** in 0.1 M Bu_4_NPF_6_/DMF.

**Figure 7:**
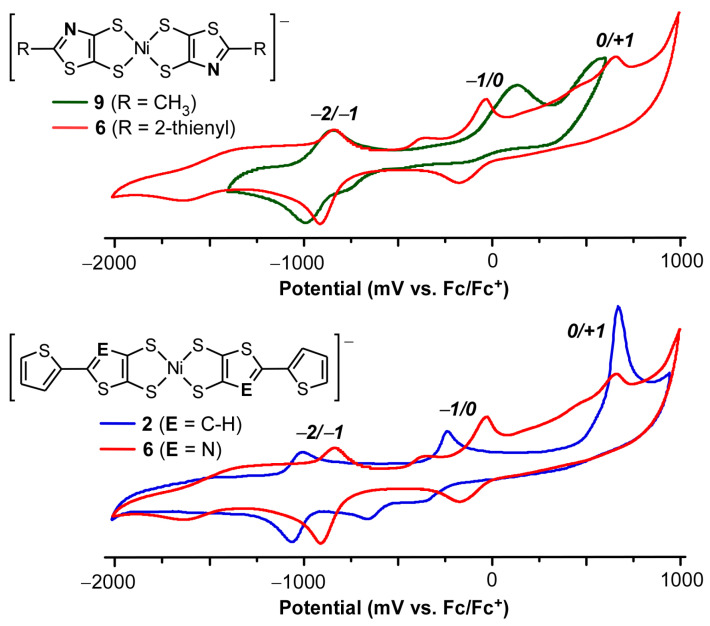
Comparative cyclic voltammograms of the thiophene-extended nickel thiazoledithiolene **6** with both the parent core **9** and the thiophenedithiolene analogue **2**, all in 0.1 M Bu_4_NPF_6_/DMF.

The nickel thiazoleditholene **9** and its aryl-extended derivatives **6**–**8** were then characterized via cyclic voltammetry in order to quantify the electronic effects of the electron-withdrawing thiazole in comparison to the thiophene of the previous nickel thiophenedithiolenes [23,24]. The collected electrochemical data are given in Table 2 and representative cyclic voltammograms are given in Figure 6 and Figure 7. As can be seen in Figure 6, the methyl-capped nickel thiazoleditholene **9** exhibits redox behavior very similar to the parent nickel thiophenedithiolene **1**. This includes two quasireversible processes corresponding to the −1/−2 and 0/−1 couples. Although the 0/−1 couple for both parent species exhibit some irreversibility, this is most likely due to some aggregation of the neutral species. As expected, the electron-withdrawing nature of thiazole shifts both the −1/−2 and 0/−1 couples of **9** to more positive potentials when compared to **1**. Thus, the −1/−2 couple shifts from an E_1/2_ of −1.09 V for **1** to −0.92 V for **9**, with similar shifts also observed for the 0/−1 couples. From the onset of the 0/−1 couples, the singly occupied molecular orbital (SOMO) energy level of the two respective monoanions can be estimated to be −4.8 eV for **1** vs. −5.0 eV for **9** [45].

An additional oxidative process corresponding to the formation of cationic species is also observed for both complexes. The formation of cationic species is somewhat atypical for most nickel dithiolene complexes and is usually only observed in complexes containing additional delocalization such as via the fused thiophene or thiazole rings here [2,6,7,9,24]. As can be seen in Figure 6, this oxidation consists of a broad and irreversible couple that occurs at about 700 mV for **1** and at slightly lower potential for **9**. As typical nickel dithiolenes do not exhibit this oxidation, it was initially thought that it may be associated with oxidation of the fused thiophene moiety of **1**, which could potentially lead to polymeric materials via oxidative polymerization [24]. However, as such oxidative coupling is not observed for either complex, this oxidation is not thought to be localized at the fused heterocycles.

As shown in Figure 7, the addition of the terminal 2-thienyl groups in compound **6** has very little effect on the potential of the −1/−2 couple. In comparison to the parent nickel thiazoledithiolene **9**, however, the 0/−1 couple for **6** occurs at less positive potential, likely due to partial delocalization of the core electron density into the peripheral aryl units. As can be seen from the data collected in Table 2, this trend is fairly consistent for all of the aryl-extended nickel thiazoledithiolenes. Finally, comparing the analogous thiophene-extended compounds **2** and **6** show nearly identical effects due to the shift from thiophenedithiolene to thiazoledithiolene as previously seen for parent compounds **1** and **9**.

### 2.4. UV-Vis-NIR Absorption Spectroscopy

**Figure 8:**
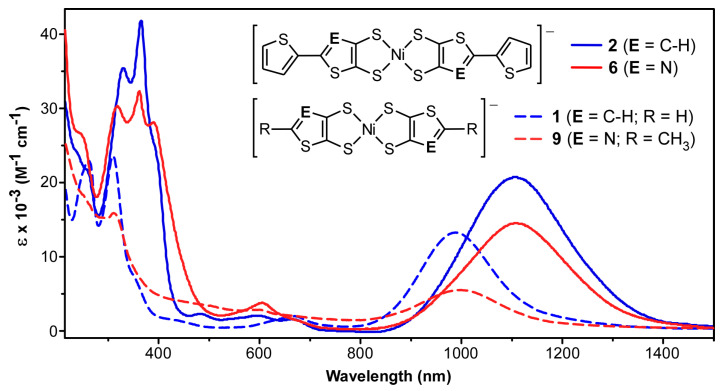
Solution absorption spectra in CH_3_CN for the parent nickel thiophenedithiolene **1** and nickel thiazoledithiolene **9** complexes in comparison to the thiophene-extended analogues **2** and **6**.

**Figure 9:**
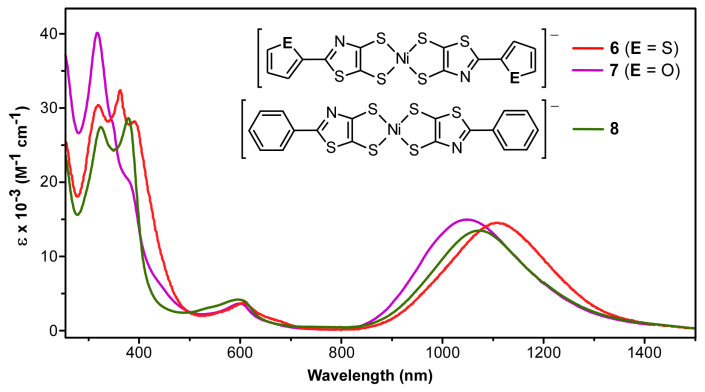
Absorption spectra of the aryl-extended nickel thiazoledithiolenes **6**–**8** in CH_3_CN.

UV-Vis-NIR spectroscopy was then used to determine any effect of heterocycle choice on the optical properties of the nickel thiazoledithiolenes in comparison to the previous nickel thiophenedithiolene analogues. The collected absorption data for the nickel thiazoledithiolenes are given in Table 3, with absorption spectra shown in Figure 8 and Figure 9. The spectra of all nickel thiophenedithiolene and thiazoledithiolene complexes are dominated by an intense NIR transition at ca. 1000 nm for the parents and ca. 1100 nm for the aryl-extended analogues. Previous analysis of monoanionic benzene-fused nickel dithiolene complexes led to an intervalence charge-transfer (IVCT) assignment for this NIR absorption, in which the electronic transition involves electron transfer from one dithiolene ligand to the other (i.e., [L•-Ni^II^-L]^−^ → [L-Ni^II^-L•]^−^) [46,47], via a SOMO-1 to SOMO transition [24]. Weaker ligand-to-metal charge-transfer (LMCT) transitions are also seen in the visible region, with more intense π → π* transitions in the UV.

As shown in Figure 8, comparisons of the parent complexes **1** and **9**, as well as the thiophene-extended complexes **2** and **6**, show that the energies of the NIR transitions are very similar, with only about a 25 nm red-shift exhibited by **9** in comparison to **1**. This energetic difference is diminished for the thiophene-extended derivatives, with both complexes exhibiting the NIR transition at ca. 1108 nm. The fact that these electronic transitions exhibit little shift in energy from thiophene to thiazole suggests that while the electron-withdrawing nature of the thiazole stabilizes the SOMO by ca. 0.2 eV, it must also stabilize the other participating molecular orbitals by a nearly equal amount [30]. Previous investigation of the solvent dependency of the NIR transition of **2** and **6** revealed very small shifts in the absorbance energy, with a corresponding bandwidth of 2000 ± 50 cm^−1^ [30]. As such, it was concluded that these electronic processes exhibited the characteristics of a fully delocalized (Class III) IVCT transition: intense absorbance (ε_max_ ≥ 5000 M^−1^ cm^−1^), narrow bandwidth (Δν_1/2_ ≤ 2000 cm^−1^) and solvent independent [48].

As can be clearly seen in Figure 8, the most significant effect of the replacement of thiophene by thiazole is that the molar absorptivities of the nickel thiazoledithiolenes are significantly diminished in comparison to the analogous nickel thiophenedithiolenes. For example, the NIR transition for the parent nickel thiazoledithiolene **9** in CH_3_CN exhibits a molar absorptivity of 5000 M^−1^ cm^−1^, nearly one-third that of the 13,700 M^−1^ cm^−1^ seen for nickel thiophenedithiolene parent **1**. This effect is lessened to some degree for the thiophene-extended complexes, with the nickel thiazoledithiolene **6** exhibiting a molar absorptivity of 14,600 M^−1^ cm^−1^, roughly two-thirds that of the thiophenedithiolene analogue **2** at 21,000 M^−1^ cm^−1^. As the NIR transitions of the thiazoledithiolenes are slightly broader than that of the thiophenedithiolenes, a better comparison would be in terms of oscillator strength (*f*). In this case, *f* = 0.14 for complex **6**, which is still less than that for complex **2** (*f* = 0.19), still corresponding to a 26% decrease in NIR absorbance for the nickel thiazoledithiolene in comparison to its thiophene analogue.

Such reduced molar absorptivity when thiophene is replaced by thiazole has been reported for a number of systems [25,26,30,49,50,51] and this difference can also be seen in the parent heterocycles themselves. Although thiophene and thiazole have very similar the absorbance energies (231 vs. 233 nm), the molar absorptivity of thiazole is only 3700 M^−1^ cm^−1^ in comparison to 7400 M^−1^ cm^−1^ for thiophene [42]. Although no significant explanation for this difference in absorptivity has been given, the molar absorptivity is related to both the allowedness of the transition and the cross-sectional area of the chromophore [52]. While it is possible that electronic differences between thiophene and thiazole could affect the transition allowedness, it is well established that the thiazole ring is smaller than thiophene [42]. As such, the cross-sectional area of thiazole analogues would be expected to be smaller, thus leading to lower absorptivity. Still, whatever the overall reasons for this reduction in molar absorptivity, it has been found that the effect is diminished in systems of greater conjugation lengths [26], as can be seen by comparing the parent complexes **1** and **9** to the thiophene-extended derivatives **2** and **6** here.

Finally, the effect of the specific aryl group on the absorption properties of the aryl-extended derivatives **6**–**8** is shown in Figure 9. In comparison to the thiophene-extended complex 6, the NIR transition of the phenyl derivative **8** is slightly blue-shifted, which agrees well with the differences seen in the corresponding thiophenedithiolenes **2** and **3**. Furthermore, this is also consistent with the fact that benzene is more aromatic than thiophene and thus benzene systems exhibit greater electron confinement and less electronic delocalization [43]. In comparison, furan is the least aromatic and thus one could conclude that the furan-extended derivative would exhibit better electron delocalization and a redshifted absorption. However, this is not the case and the NIR transition of **7** is observed at even higher energy than the phenyl derivative **8**. However, such a blue-shift in absorbance caused by replacing thiophene with furan is consistent with various previous reports of conjugated systems [53,54,55].

### 2.5. Theoretical Methods

To further investigate the optical and electronic properties of the nickel thiazoledithiolenes, DFT methods were used to calculate the relative energies and molecular orbital (MO) electronic density contours for the parent nickel thiazoledithiolene and the aryl-extended derivatives **6**–**8**. Methods used both the B3LYP-D3 and CAM-B3LYP-D3 functionals, with the CAM-B3LYP-D3 functional found to provide improved quantitative agreement with the experimental data compared to the B3LYP-D3 functional. As such, the CAM-B3LYP-D3 results are discussed here, with the additional B3LYP-D3 results given in the SI. In all cases, the lowest energy structures of the dithiolene complexes were found to be completely planar.

**Figure 10:**
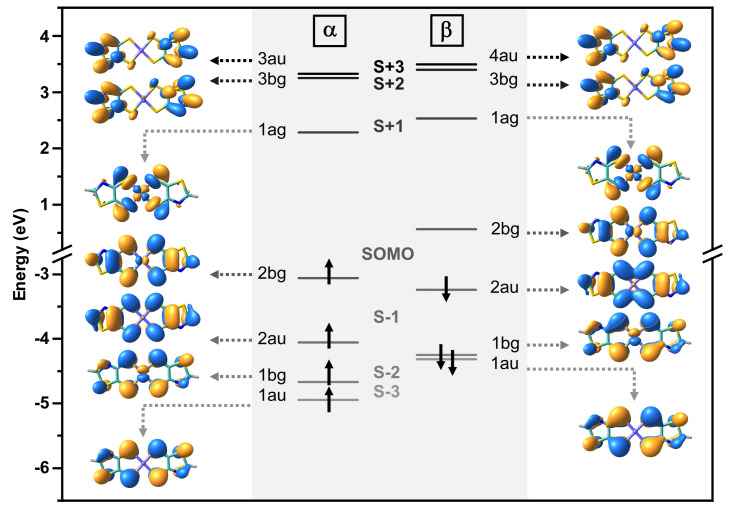
Electronic density contours and energy levels of the MOs of the core nickel thiazoledithiolene (NiTzDT), calculated at the CAM-B3LYP-D3/def2-TZVP level of theory.

The electronic density contour for the SOMO of the core nickel thiazoledithiolene (NiTzDT) is shown in Figure 10, along with other MOs of relatively close energetic spacing. As can be seen, the SOMO of the monoanion consists of a mixture of ligand-metal orbitals, although primarily of ligand character. The general contour of the SOMO is in good agreement with that of other monoanionic nickel dithiolenes [24,56], as well as the lowest unoccupied MO of previously calculated neutral nickel dithiolenes [2].

**Figure 11:**
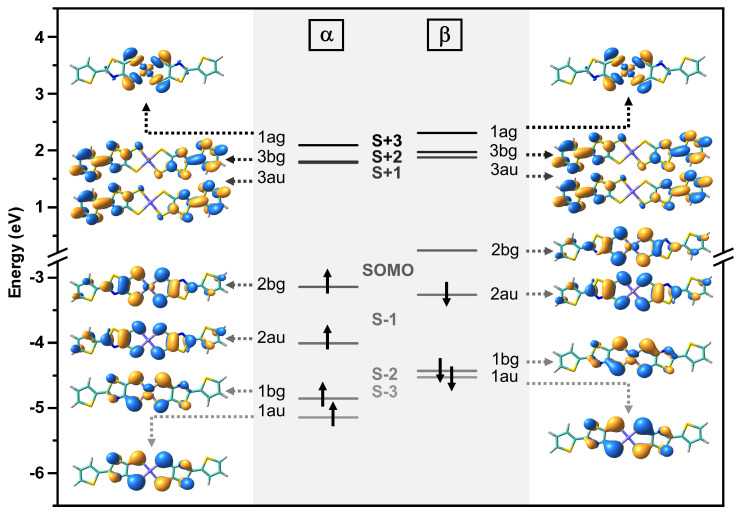
Electronic density contours and energy levels of the MOs of the thiophene-extended complex **6,** calculated at the CAM-B3LYP-D3/def2-TZVP level of theory.

In order to evaluate the participation of the aryl-extension, the electronic density contour for the SOMO and associated MOs of the thiophene-extended nickel thiazoledithiolene 6 is shown in Figure 11. As can be seen, the general contour of the SOMO is very similar to that shown in Figure 10 for parent, but now also shows some electron density extending out onto the pendent thiophene as well. The general contour and additional delocalization also agree well with the analogous nickel thiophenedithiolene **2** as previously reported [24]. In addition, much greater delocalization towards the molecular periphery can be seen in some of the other MOs, particularly the SOMO+1 and SOMO+2, further confirming the electronic contribution of the pendent aryl groups in the aryl-extended derivatives.

The vertical excitation energies were then calculated using the time-dependent DFT (TDDFT) approach, with the collected results via CAM-B3LYP-D3 functional given in Table 4. The trends in transition energies agree fairly well with the experimental data, although the calculated NIR transitions are red shifted by 70 nm or more. The primary outlier is the furan-extended complex **7**, which experimentally exhibits a ca. 60 nm blue shift relative to the thiophene-extended complex **6**, yet the calculated value is instead red shifted by ca. 300 nm. The oscillator strengths are also significantly overestimated in all cases.

As with the experimental spectra, the low-energy excitations are dominated by an NIR transition that is attributed to a one-electron excitation from the SOMO-1 to the SOMO, which is in good agreement with previous analyses [24,46,47]. Although the calculated energies of the NIR transition occur at lower energies than those determined by experimental absorption spectra, this is minimized in the case of the core nickel thiazoledithiolene NiTzDT, with a difference of ca. 70 nm. However, thinking about this on a linear scale, this corresponds to only a difference of 0.07 eV.

In the case of the aryl-extended derivatives, the difference increases to ca. 95–110 nm. However, some of the increase here is due to the fact that the calculated energies are based on the fully planar confirmations, while the experimental measurements are of the complexes in solution. As such, it is expected that some rotation of the interannular bond between the dithiolene core and the exterior aryl groups occur to allow a better match of the compound polarity with that of the solvent. Such deviations from planarity would thus reduce conjugation and increase the energy of the experimentally determined transitions, with this increased energy likely responsible for the increased energetic differences between experimental and theory for these aryl-extended derivatives.

### 2.6. Comparison of the Phenyl-Extended Complex ***8*** with the Previous Work of Kibbel

Finally, it is worth comparing this work with that previously reported by Kibbel and coworkers [27]. As pointed out in the introduction, the phenyl-extended complex **8** reported here should technically be identical to the previously reported monoanion **4a**. However, these compounds have been given different compound numbers to better clarify that they are prepared by different methods and to better allow the direct comparison of these two species. Of course, a complication is that Kibbel and coworkers did not really report anything to confirm the identity of their reported complexes, giving only melting points and a statement that elemental analyzes corresponded to the calculated values [27]. In terms of the melting points, compound **8** here was found to have a melting point range of 181.0–181.7 °C, while Kibbel and coworkers reported a range of 207–208 °C for **4a**. It is important to point out that both complexes are monoanionic salts of the same Bu_4_N^+^ cation and that the melting point reported for **4a** is higher than that found for any of the aryl-extended nickel thiazoledithiolenes **6**–**8**. However, the value given for **4a** is consistent with the other analogous monoanions reported by Kibbel and coworkers [27].

Other specific data that can be compared are the electrochemical potentials of the two species. While the values reported by Kibbel and coworkers were measured using a different electrolytic solution and against a different reference electrode, adjusting the potentials for the difference in reference gives a value of −0.89 V (vs. ferrocene) for the −1/−2 couple of **4a**, which agrees well with the value of −0.90 V for the −1/−2 couple of **8**. There is a greater difference in the values of the 0/−1 couples, but this could be due to the differences in electrolytes used. Furthermore, it is stated that this second couple causes deposition of an insoluble product on the surface of the electrode, thus reducing the accuracy of the value [27].

Lastly, a qualitative description of the UV-vis spectrum of **4a** is also given, which describes two bands in the UV at 240–360 nm, followed by two additional bands at 370–390 and 590–615 nm [27]. Unfortunately, no information is given about the relative intensity of these bands, but a glaring issue is the lack of the distinctive NIR transition of the nickel thiazoledithiolenes. One possibility is that the instrument used did not have the capability to measure absorption at wavelengths greater than 800 nm, but as no experimental details are given, this is unknown. The other possibility is that **4a** is not the complex it is thought to be.

One possible explanation that could account for the differences in the characterization of these two species is that **4a** is not the same complex as **8**, but is rather the corresponding sulfur-oxidized thiazoledithiolene complex similar to **6**-O_4_ described above. As previously shown, such sulfur-oxidized species can be easily formed for the thiazoledithiolenes depending on the exact details of the O_2_ oxidation of the originally formed dianion complex [30]. As the only data that really agrees between the two reports are the redox couples, this could be due to the use of the analogous dianion for the electrochemical measurements, as both the dianion and monoanion should result in the same electrochemistry. Again, however, no experimental details were reported and thus it is unknown which of the two isolated complexes was used for this characterization. As the sulfur-oxidized analogue would be higher molecular weight, this could then account for the higher melting point and previous characterization of the sulfur-oxidized species **6**-O_4_ confirmed a prominent visible transition near 600 nm, but no NIR transition for such species.

## 3. Materials and Methods

Tetrabutylammonium bis(thiophenedithiolato)nickelate(1−) ([Bu_4_N][**1**]) [24], tetrabutylammonium bis[2-(2-thienyl)-4,5-thiophenedithiolato]nickelate(1−) ([Bu_4_N][**2**]) [24], tetrabutylammonium bis[2-phenyl-4,5-thiophenedithiolato]nickelate(1−) ([Bu_4_N][**3**]) [24], 2,4,5-tribromothiazole (**10**) [32], and 4,5-dibromothiazole (**17**) [32] were prepared as previously reported. ZnCl_2_ was dried in vacuo prior to use. Dry diethyl ether and THF were obtained via distillation over sodium/benzophenone. DMF used for the electrochemical measurements was dried with MgSO_4_ and filtered through a plug of silica gel. All other chemical species were reagent grade and used without further purification. All reactions were carried out under an inert atmosphere. All glassware for chemical synthesis was oven-dried, assembled while still hot, and cooled under dry nitrogen. Chromatography was performed using standard methods with 230–400 mesh silica gel. Melting points were obtained with a digital thermocouple accurate to 0.1 °C resolution. HRMS (ESI-TOF) was performed in-house and ^1^H and ^13^C NMR spectra were collected on a 400 MHz spectrometer (400 MHz ^1^H, 100 MHz ^13^C) in CDCl_3_ and referenced to the CHCl_3_ signal at 7.26 ppm. Peak multiplicity is reported as follows: s = singlet, dd = doublet of doublets, m = multiplet.

### 3.1. General Procedure for Synthesis of 2-Aryl-4,5-dibromothiazoles

A solution of thiophene, furan, or bromobenzene (12 mmol) in 150 mL diethyl ether was cooled to 0 °C. BuLi (4.8 mL, 2.5 M in hexanes, 12 mmol) was then added dropwise, and the solution allowed to stir 30 min. ZnCl_2_ (1.640 g, 12 mmol) was added and the solution stirred for 30 min at 0 °C before warming to ambient temperature. The solution was stirred for an additional 1 h at room temperature, becoming opaque and white. 2,4,5-Tribromothiazole (3.240 g, 10 mmol) and Pd(dppf)Cl_2_ (0.184 g, 2.5 mol%) were added and the mixture was stirred for 3 h. Aqueous NaHCO_3_ was added to quench the reaction, and the organic layer was separated, after which the remaining aqueous layer was extracted with diethyl ether (100 mL). The organic fractions were combined, dried with MgSO_4_, filtered, and concentrated in vacuo. The crude product was then purified by silica gel column chromatography using 5% diethyl ether in hexanes.

#### 3.1.1. 4,5-Dibromo-2-(2-thienyl)thiazole (**11**)

Isolated as a faintly yellow solid (60–66% yield). mp 73.4–74.6 °C (lit. mp 73.4–74.6 °C [30]). ^1^H NMR: δ 7.45 (dd, *J* = 3.8, 1.1 Hz, 1H), 7.44 (dd, *J* = 5.1, 1.1 Hz, 1H), 7.08 (dd, *J* = 5.1, 3.8 Hz, 1H). ^13^C NMR: δ 162.7, 135.8, 129.1, 128.9, 128.1, 127.3, 105.7. HRMS: m/z 325.8276 [M + H]^+^ (calcd for C_7_H_4_Br_2_NS_2_ 325.8255). NMR data agree well with previously reported values [30].

#### 3.1.2. 4,5-Dibromo-2-(2-furyl)thiazole (**12**)

Isolated as a white solid (62–65% yield). mp 98.9–99.8 °C. ^1^H NMR: δ 7.51 (dd, *J* = 1.7, 0.6 Hz, 1H), 7.05 (dd, *J* = 3.5, 0.6 Hz, 1H), 6.55 (dd, *J* = 3.5, 1.7 Hz, 1H). ^13^C NMR: δ 158.8, 147.6, 144.4, 129.4, 112.6, 110.1, 106.2. HRMS: m/z 309.8362 [M + H]^+^ (calcd for C_7_H_4_Br_2_NOS 309.8360).

#### 3.1.3. 4,5-Dibromo-2-phenylthiazole (**13**)

Isolated as a white solid (50–54% yield). mp 62.1–63.5 °C. ^1^H NMR: δ 7.85 (m, 2H), 7.45 (m, 3H). ^13^C NMR: δ 169.3, 132.3, 131.1, 129.2, 127.2, 126.1, 106.8. HRMS: m/z 319.8573 [M + H]^+^ (calcd for C_9_H_6_Br_2_NS 319.8567).

### 3.2. 4,5-Dibromo-2-methylthiazole *(**20**)*

Mg (0.291 g, 12 mmol) and a single crystal of iodine were added to a 125 mL oven-dried, round-bottom flask and purged with nitrogen gas. Diethyl ether (50 mL) was then added and CH_3_I (0.40 mL, 6 mmol) was added dropwise over the course of 30 min, with the solution becoming cloudy due to the formation of a white precipitate. The reaction was allowed to stir for 1 h after addition was complete and cloudy suspension subsequently transferred via cannula to a solution of 2,4,5-tribromothiazole (1.640 g, 5 mmol), CuO (0.397 g, 5 mmol), and Ni(dppp)Cl_2_ (0.135 g, 5 mol%) in 100 mL diethyl ether. The mixture was stirred overnight at ambient temperature, after which saturated NaHCO_3_ was added. The organic layer was then separated and the remaining aqueous layer extracted with diethyl ether. The organic fractions were combined, dried over MgSO_4_, filtered, concentrated in vacuo, and chromatographed on silica gel to afford a clear-yellow oil (73% yield). ^1^H NMR: δ 2.67 (s, 3H). ^13^C NMR: δ 167.5, 127.5, 105.7, 19.9. HRMS: m/z 257.8436 [M + H]^+^ (calcd for C_4_H_4_Br_2_NS 257.8410).

### 3.3. General Procedure for Synthesis of Acetyl-Protected Ligands

The desired 2-aryl-4,5-dibromothiazole (5.0 mmol) was added to a 250 mL round-bottom flask, which was then evacuated and backfilled with nitrogen. Diethyl ether (100 mL) was added and the solution cooled to −78 °C. *tert*-Butyllithium (3.2 mL, 1.7 M in pentane, 5.5 mmol) was then added via metal syringe and the solution stirred for 1 h. Sulfur (0.16 g, 5.0 mmol) was added, and the solution stirred for an additional 1 h. In a separate 500 mL round-bottom flask, 150 mL diethyl ether was added and cooled to −78 °C, after which butyllithium (5.0 mL, 2.5 M in hexanes, 12.5 mmol) was added. Once this second solution was prepared, the initial solution was warmed to room temperature and transferred via cannula into the new solution of butyllithium. The combined solution was stirred for 2 h, after which sulfur (0.40 g, 12.5 mmol) was added and stirring continued for an additional 1 h. The solution was then warmed to ambient temperature, resulting in the formation of a precipitate. The solution was then cooled back to −78 °C, after which acetyl chloride (2.1 mL, 30 mmol) was added and the mixture stirred for 15 min. The reaction was warmed to room temperature, poured into saturated aqueous NaHCO_3_ (150 mL), and the organic layer separated. The remaining aqueous layer was then extracted with diethyl ether (100 mL) and the combined organic layers dried with MgSO_4_, filtered, and concentrated in vacuo, resulting in a strongly odiferous, oily product. The crude oil was then purified by silica gel column chromatography using a mixture of 5% ethyl acetate in hexanes.

#### 3.3.1. 4,5-Bis(thioacetate)-2-(2-thienyl)thiazole (**14**)

Isolated as a yellow solid (30–33% yield). mp 100.8–102.1 °C. ^1^H NMR: δ 7.56 (dd, *J* = 3.7, 1.1 Hz, 1H), 7.45 (dd, *J* = 5.1, 1.1 Hz, 1H), 7.09 (dd, *J* = 5.1, 3.7 Hz, 1H), 2.48 (s, 3H), 2.44 (s, 3H). ^13^C NMR: δ 191.7, 190.8, 169.6, 165.2, 144.8, 129.1, 128.1, 127.8, 127.6, 30.3, 30.0. HRMS: m/z 315.9621 [M + H]^+^ (calcd for C_11_H_10_NO_2_S_4_ 315.3594). NMR data agree well with previously reported values [30].

#### 3.3.2. 4,5-Bis(thioacetate)-2-(2-furyl)thiazole (**15**)

Isolated as a yellow solid (30–35% yield). ^1^H NMR: δ 7.55 (dd, *J* = 0.7, 1.8 Hz, 1H), 7.09 (dd, *J* = 0.7, 3.4 Hz, 1H), 6.55 (dd, *J* = 1.8, 3.4 Hz, 1H), 2.45 (s, 3H), 2.41 (s, 3H). ^13^C NMR: δ 191.7, 190.7, 161.3, 148.1, 145.3, 144.5, 127.6, 112.5, 110.5, 30.3, 30.0. HRMS: m/z 247.9821 [M + H]^+^ (calcd for C_11_H_10_NO_3_S_3_ 299.9823).

#### 3.3.3. 4,5-Bis(thioacetate)-2-phenylthiazole (**16**)

Isolated as an off-white solid (29–32% yield). mp 104.1–105.3 °C. ^1^H NMR: δ 7.92 (m, 2H), 7.44 (m, 3H), 2.48 (s, 3H), 2.45 (s, 3H). ^13^C NMR: δ 191.9, 190.8, 171.7, 145.4, 132.7, 131.0, 129.1, 129.0, 128.3, 126.6, 126.4, 30.3, 30.0. HRMS: m/z 310.0007 [M + H]^+^ (calcd for C_13_H_12_NO_2_S_3_ 310.0030).

#### 3.3.4. 4,5-Bis(thioacetate)-2-methylthiazole (**21**)

Isolated as a white solid (31–36% yield). mp 61.5–63.0 °C. ^1^H NMR: δ 2.75 (s, 3H), 2.45 (s, 3H), 2.42 (s, 3H). ^13^C NMR: δ 192.0, 190.9, 170.5, 144.2, 127.8, 30.2, 29.9, 19.8. HRMS: m/z 247.9869 [M + H]^+^ (calcd for C_8_H_10_NO_2_S_3_ 247.9874).

### 3.4. General Procedure for Synthesis of Nickel Thiazoledithiolenes

A 125 mL round bottom flask was filled with 100 mL of methanol and deoxygenated via sequential freeze-pump-thaw cycles. Once the methanol had completely thawed at the end of the final freeze-pump-thaw cycle, sodium metal (4.00 g) was added to the still cold solution (ca. −90 °C) and the mixture allowed to react for 1 h, during which the solution warmed to room temperature. The desired acetyl-protected ligand (0.64 mmol) was then added to the sodium methoxide solution and stirred for 1 h. A separate solution of Ni(H_2_O)_6_Cl_2_ (0.076 g, 0.32 mmol) in 5 mL of nitrogen-purged methanol was then added dropwise, prompting a color change from yellow to red. The mixture was allowed to stir for 45 min, after which tetrabutylammonium bromide (Bu_4_NBr, 0.820 g, 2.54 mmol) was added. Finally, the solution was opened to ambient atmosphere and 50 mL of non-deoxygenated H_2_O was added to prompt precipitation. The precipitate was collected by filtration and washed sequentially with H_2_O, methanol, and diethyl ether. The precipitate was dissolved in MeCN and recrystallized using diethyl ether as the countersolvent.

#### 3.4.1. Tetrabutylammonium Bis[2-(2-thienyl)-4,5-thiazoledithiolato]nickelate(1−) ([Bu4N][**6**])

Isolated as a dark green crystalline solid (40–43% yield). mp 146.4–146.6 °C (lit. mp 146.6–146.8 °C [30]). HRMS: m/z 515.7669 [M−] (calcd for C_14_H_6_N_2_NiS_8_ 515.7650). Anal. Calc. (C_14_H_6_N_2_NiS_8_ ‧ 0.25 CHCl_3_): C 46.36, H 5.40, N 5.36 (%); Found: C 46.54, H 5.00, N 5.12 (%).

#### 3.4.2. Tetrabutylammonium Bis[2-(2-furyl)-4,5-thiazoledithiolato]nickelate(1−) ([Bu4N][**7**])

Isolated as a dark green crystalline solid (37–42% yield). HRMS: m/z 483.8123 [M−] (calcd for C_14_H_6_N_2_O_2_NiS_6_ 483.8107).

#### 3.4.3. Tetrabutylammonium Bis(2-phenyl-4,5-thiazoledithiolato)nickelate(1−) ([Bu4N][**8**])

Isolated as a dark green crystalline solid (36–40% yield). mp 181.0–181.7 °C. HRMS: m/z 503.8534 [M−] (calcd for C_18_H_10_N_2_NiS_6_ 503.8522).

#### 3.4.4. Tetrabutylammonium Bis(2-methyl-4,5-thiazoledithiolato)nickelate(1−) ([Bu4N][**9**])

Isolated as a dark violet crystalline solid (16–22% yield). mp 126.5–127.2 °C. HRMS: m/z 379.8221 [M−] (calcd for C_8_H_6_N_2_NiS_6_ 379.8209).

### 3.5. X-Ray Crystallography

X-ray quality crystals of [Bu_4_N][**8**] were grown via vapor diffusion, with CH_3_CN as the solvent and diethyl ether as the antisolvent. The X-ray intensity data of the crystals were measured at 100 K on a Bruker Kappa Apex II Duo CCD-based X-ray diffractometer system equipped with a Mo-target X-ray tube (λ = 0.71073 Å) operated at 2000 W of power. The detector was placed at a distance of 5.000 cm from the crystal and data collected via the Bruker APEX2 software package. The frames were integrated with the Bruker SAINT software package. The unit cell was determined and refined by least-squares upon the refinement of XYZ-centeroids of reflections above 20σ(I). The structure was refined using the Bruker SHELXTL (Version 5.1) Software Package.

Crystal Data for [Bu_4_N][**8**] (C_34_H_46_N_3_NiS_6_, M = 746.14 g/mol): monoclinic, space group P2_1_, a = 9.3688(3) Å, b = 19.0354(6) Å, c = 10.4650(4) Å, α = 90°, β = 107.512(2)°, γ = 90°, *V* = 1779.82(11) Å^3^, *Z* = 2, T = 102(2) K, μ = 4.291 mm^−1^, D_calc_ = 1.395 g/cm^3^, 6274 reflections measured, 5597 unique (R_int_ = 0.0591) which were used in all calculations. The final R_1_ was 0.0408 (I > 2σ(I)) and wR_2_ was 0.1048 (all data).

CCDC 2477645 contains the supplementary crystallographic data for this paper. These data can be obtained free of charge via https://www.ccdc.cam.ac.uk/structures/, accessed on 5 October 2025.

### 3.6. Theoretical Methodology

All theoretical calculations were performed in the gas phase using Gaussian 16 package [57]. Simulations were carried out within the framework of the density functional theory (DFT) using the long-range corrected hybrid CAM-B3LYP [58] functional together with def2-TZVP [59,60] basis set. An empirical dispersion correction term, as proposed by Grimme [61], was included via D3.

All molecular structure simulations were carried out on their radical anion forms, by using an unrestricted wavefunction and applying symmetry constraints corresponding to the C_2h_ point group.

Firstly, the minimum molecular geometries were fully optimized. The absence of imaginary frequencies ensured that the global minimum energy was found. Then, using the time-dependent DFT (TD-DFT) [62,63] approach, we computed the vertical electronic excitation energies on the previously optimized molecular geometries. Molecular orbitals distribution were plotted using the Chemcraft 1.8 molecular modelling software [64].

We also used the generalized gradient approximation (GGA) functional B3LYP [65,66], together with the same def2-TZVP basis set and D3 dispersion correction, in order to evaluate the molecular orbitals distribution and the optical trends. In this case, CAM-B3LYP-D3 functional is found to provide an improved quantitative agreement with the experimental data compared to B3LYP-D3 functional.

### 3.7. Absorption Spectroscopy

Absorption spectroscopy was performed on a dual-beam scanning UV-vis-NIR spectrophotometer using samples prepared as dilute CH_3_CN solutions in matching 1 cm quartz cuvettes. Extinction coefficients were calculated via Beer’s law, using the adsorption values of 3–5 different micromolar solutions. Oscillator strengths were determined from the visible spectra via spectral fitting to accurately quantify the area of each transition and then calculated using literature methods [67].

### 3.8. Electrochemistry

All electrochemical methods were performed utilizing a three-electrode cell consisting of platinum disc working electrode, a platinum wire auxiliary electrode, and a Ag/Ag^+^ reference electrode (0.251 V vs. SCE) [68]. Supporting electrolyte consisted of 0.10 M tetrabutylammonium hexafluorophosphate (Bu_4_NPF_6_) in dry DMF. Solutions were deoxygenated by sparging with argon prior to each scan and blanketed with argon during the measurements. All measurements were collected at a scan rate of 100 mV/s and potentials were referenced vs. an internal ferrocene standard (50 mV vs. Ag/Ag^+^). Energy values for the compound SOMOs were estimated from the onsets of the oxidation of the monoanion in relation to ferrocene, using the value of 5.1 eV vs. vacuum for ferrocene [45].

## 4. Conclusions

A series of diaryl-capped Ni thiazoledithiolene complexes have been synthesized and characterized in terms of their optical and electronic properties. In addition, the dimethyl-capped complex has also been prepared as a model of the simple Ni thiazoledithiophene core. Of particular interest was the direct comparison of these Ni thiazoledithiolene complexes with their Ni thiophenedithiolene analogues in order to determine the extent of stabilization of the SOMO and any potential reduction in absorptivity as previously found for other thiazole-based systems. In terms of the SOMO stabilization, both the core Ni thiazoledithiolene and the aryl-extended derivatives exhibited a stabilization of ca. 0.2 eV in comparison to the thiophenedithiolene analogues. The energy of the characteristic NIR transition shows only minor shifts between the thiazole and thiophene systems, which suggests that while the electron-withdrawing nature of the thiazole stabilizes the SOMO by ca. 0.2 eV, it must also stabilize the other participating molecular orbitals by a nearly equal amount. In terms of any differences in the extent of visible light absorption, it was found that the core Ni thiazoledithiolene complex exhibited a 62% reduction in absorption in comparison to the thiophenediolene complex. However, this was found to drop to only a 26% reduction for the corresponding aryl-extended derivatives, which agrees with previous studies that revealed the negative effect of the thiazole on absorption coefficients is minimized by extending the conjugation of the system. Overall, thiazoledithiolenes offer significant stabilization of the frontier orbitals without significant changes in the energy of the NIR absorption, and only a small loss in absorptivity in comparison to it thiophene analogues. As such, it offers significant promise for the electronic tuning of these species for potential applications in technological devices.

## Figures and Tables

**Table 1:** Selected bond lengths [Å] of nickel thiazoledithiolenes **6** and **8**, along with thiazole and nickel thiophenedithiolenes **2** and **3** for comparison.

Parameter	Thiazole ^1^	[Bu_4_N][6] ^2^	[Bu_4_N][8]	[NMP][2] ^3^	[NMP][3] ^4^
Ni-S1		2.1762(9)	2.172(1)	2.175(2)	2.174(3)
Ni-S2		2.1656(9)	2.170(2)	2.170(2)	2.176(3)
S1-C1		1.733(3)	1.739(6)	1.708(6)	1.732(9)
S2-C2		1.717(3)	1.712(5)	1.733(7)	1.731(10)
C1-C2	1.367	1.376(4)	1.371(7)	1.396(8)	1.442(12)
C2-S3	1.713	1.728(3)	1.732(6)	1.748(6)	1.725(8)
C1-N1	1.372	1.378(4)	1.373(7)		
N1-C3	1.304	1.310(4)	1.306(7)		
S2-C3	1.724	1.754(3)	1.752(5)	1.746(6)	1.739(8)
C3-C4		1.441(4)	1.469(8)	1.472(8)	1.51(1)

^1^ Ref. [42]. ^2^ Ref. [30]. ^3^ Ref. [23], NMP = *N*-methylpyridinium. ^4^ Ref. [24], NMP = *N*-methylpyridinium.

**Table 2:** Electrochemical Data for Nickel Thiazoledithiolene Complexes.

Compound	*E*_1/2_^2−/1−^ (V) ^1^	Δ*E* (mV)	*E*_1/2_^0/1−^ (V) ^1^	Δ*E* (mV)	*E*_p_^0/n+^ (V) ^1^
[Bu_4_N][**9**]	−0.92	150	0.01	250	0.58
[Bu_4_N][**6**]	−0.88	80	−0.10	120	0.67
[Bu_4_N][**7**]	−0.87	180	−0.05	250	0.85
[Bu_4_N][**8**]	−0.90	100	−0.18	200	0.65

^1^ All potentials versus Fc/Fc^+^ (0.1 M Bu_4_NPF_6_/DMF solution).

**Table 3:** UV-Vis-NIR Absorption Data for Nickel Thiazoledithiolene Complexes in CH_3_CN.

Compound	λ_max_ (nm)	ε (M^−1^ cm^−1^)	*f*	Assignment
[Bu_4_N][**9**]	1014	5000	0.05	IVCT
	295	16,000	0.10	π → π*
[Bu_4_N][**6**]	1108	14,600	0.14	IVCT
	610	3800	0.06	LMCT
	365	32,500	0.60	π → π*
[Bu_4_N][**7**]	1051	15,000	0.15	IVCT
	605	3600	0.06	LMCT
	316	40,000	0.62	π → π*
[Bu_4_N][**8**]	1072	13,500	0.12	IVCT
	600	4100	0.06	LMCT
	377	38,800	0.52	π → π*

**Table 4:** TDDFT-calculated vertical excitation energies at CAM-B3LYP-D3/def2-TZVP level of theory.

Compound	λ_max_^calc^ (nm)	*f* ^calc^	Description	Assignment
NiTzDT	1082	0.14	SOMO-1β → SOMOβ (97%)	IVCT
	612	0.02	SOMO-3β → SOMOβ (96%)	LMCT
	297	0.09	SOMOα → SOMO+3α (17%)	MLCT
			SOMO-3β → SOMO+2β (15%)	π → π*
			SOMO-1β → SOMO+2β (14%)	π → π*
			SOMO-2β → SOMO+3β (13%)	MLCT
	270	0.36	SOMO-4α → SOMO+1α (16%)	π → π*
			SOMO-5β → SOMO+1β (25%)	π → π*
			SOMO-7β → SOMO+1β (15%)	π → π*
**6**	1203	0.28	SOMO-1β → SOMOβ (96%)	IVCT
	616	0.02	SOMO-3β → SOMOβ (91%)	LMCT
	590	0.06	SOMOα → SOMO+1α (36%)	MLCT
			SOMO-1α → SOMO+2α (21%)	π → π*
			SOMO-1β → SOMO+2β (17%)	π → π*
	389	0.64	SOMOα → SOMO+1α (32%)	MLCT
			SOMO-1β → SOMO+2β (35%)	π → π*
	283	0.31	SOMO-2α → SOMO+1α (28%)	MLCT
			SOMO-3α → SOMO+2α (22%)	π → π*
	253	0.21	SOMO-4α → SOMO+3α (24%)	π → π*
			SOMO-5β → SOMO+3β (31%)	π → π*
**7**	1341	0.26	SOMO-1β → SOMOβ (96%)	IVCT
	631	0.02	SOMO-3β → SOMOβ (89%)	LMCT
	590	0.06	SOMOα → SOMO+1α (35%)	MLCT
			SOMO-1α → SOMO+2α (22%)	π → π*
			SOMO-1β → SOMO+2β (19%)	π → π*
	382	0.71	SOMOα → SOMO+1α (32%)	MLCT
			SOMO-1β → SOMO+2β (36%)	π → π*
	302	0.45	SOMO-8β → SOMOβ (52%)	LMCT
	257	0.19	SOMO-4α → SOMO+3α (20%)	π → π*
			SOMO-5β → SOMO+3β (25%)	π → π*
			SOMO-7β → SOMO+3β (20%)	MLCT
**8**	1184	0.26	SOMO-1β → SOMOβ (96%)	IVCT
	618	0.02	SOMO-3β → SOMOβ (93%)	LMCT
	551	0.06	SOMOα → SOMO+1α (37%)	MLCT
			SOMO-1α → SOMO+2α (21%)	π → π*
			SOMO-1β → SOMO+2β (18%)	π → π*
	375	0.71	SOMOα → SOMO+1α (31%)	MLCT
			SOMO-1β → SOMO+2β (42%)	π → π*
	278	0.45	SOMO-2α → SOMO+1α (19%)	MLCT
	252	0.19	SOMO-3α → SOMO+2α (13%)	π → π*
			SOMO-4α → SOMO+3α (23%)	π → π*
			SOMO-5β → SOMO+3β (29%)	π → π*

## Data Availability

The data presented in this study are available either within the article or the associated Supplementary Material.

## Supplementary Materials

The following supporting information can be downloaded at: https://www.mdpi.com/article/10.3390/molecules30193998/s1, Figures S1–S12: ^1^H NMR and ^13^C NMR spectra; Figure S13: Crystal packing of [Bu_4_N][**8**]; Figure S14: Electronic density contours of the MOs of the core nickel thiazoledithiolene (NiTzDT), calculated at the CAM-B3LYP-D3/def2-TZVP level of theory; Figure S15: Electronic density contours of the MOs of the thiophene-extended complex **6**, calculated at the CAM-B3LYP-D3/def2-TZVP level of theory; Figure S16: Electronic density contours of the MOs of the furan-extended complex **7**, calculated at the CAM-B3LYP-D3/def2-TZVP level of theory; Figure S17: Electronic density contours of the MOs of the phenyl-extended complex **8**, calculated at the CAM-B3LYP-D3/def2-TZVP level of theory; Table S1: TDDFT-calculated vertical excitation energies at UB3LYP-D3/def2-TZVP level of theory; Figure S18: Electronic density contours of the MOs of the core nickel thiazoledithiolene (NiTzDT), calculated at the UB3LYP-D3/def2-TZVP level of theory; Figure S19: Electronic density contours of the MOs of the thiophene-extended complex **6**, calculated at the UB3LYP-D3/def2-TZVP level of theory; Figure S20: Electronic density contours of the MOs of the furan-extended complex **7**, calculated at the UB3LYP-D3/def2-TZVP level of theory; Figure S21: Electronic density contours of the MOs of the phenyl-extended complex **8**, calculated at the UB3LYP-D3/def2-TZVP level of theory.

## Abbreviations

The following abbreviations are used in this manuscript:

BuLi	butyllithium
CCDC	Cambridge Crystallographic Data Centre
CCD	charge-coupled device
DFT	density functional theory
DMF	dimethylformamide
ESI-TOF	electrospray ionization time-of-flight
HRMS	high resolution mass spectrometry
IVCT	intervalence charge-transfer
LMCT	ligand-to-metal charge-transfer
MBDT	metal benzenedithiolene
MDT	metal dithiolene
MTDT	metal thiophenedithiolene
MO	molecular orbital
NIR	near infra-red
NiTzDT	nickel thiazoledithiolene
NMP	*N*-methylpyridinium
NMR	nuclear magnetic reasonance
*f*	oscillator strength
SCE	saturated calomel electrode
SOMO	singly occupied molecular orbital
*^t^*BuLi	*tert*-butyllithium
Bu_4_N	tetrabutylammonium
THF	tetrahydrofuran
TDDFT	time dependent density functional theory
TIPS	triisopropylsilyl
UV	ultraviolet
