# Supplementary material for: Nickel Thiazoledithiolenes: π-Extended Fused-Ring Metal Dithiolenes as Highly Delocalized π-Electron Systems with Stabilized Frontier Orbitals
Source: Molecules. 2025 Oct 6;30(19):3998. doi: 10.3390/molecules30193998 (PMC12525549; doi:10.3390/molecules30193998)
Supplement: Supplementary file 1 [file molecules-30-03998-s001.zip › molecules-3866216-supplementary.pdf]

Molecules **2025**, *30*, 3998

Nickel Thiazoledithiolenes: π-Extended Fused-Ring Metal Dithiolenes as Highly Delocalized π-Electron Systems with Stabilized Frontier Orbitals

Eric J. Uzelac, Juan Sánchez-Rincón, M. Carmen Ruiz Delgado, and Seth C. Rasmussen*

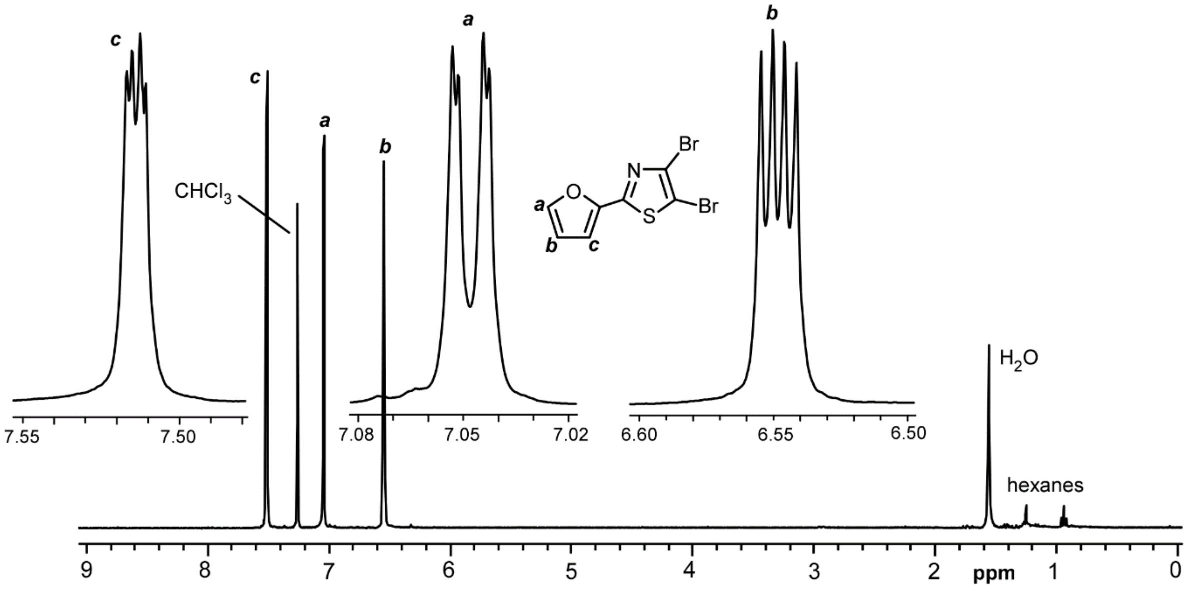

Figure S1. ^{1}H NMR Spectrum of 4,5-dibromo-2-(2-furyl)thiazole (**12**).

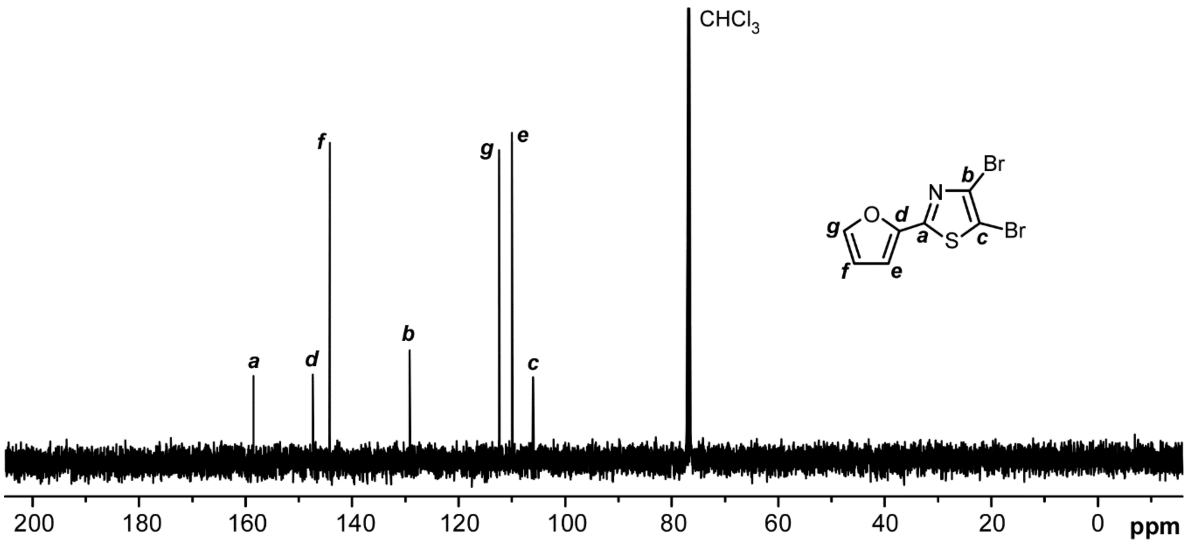

Figure S2. ^{13}C NMR Spectrum of 4,5-dibromo-2-(2-furyl)thiazole (**12**).

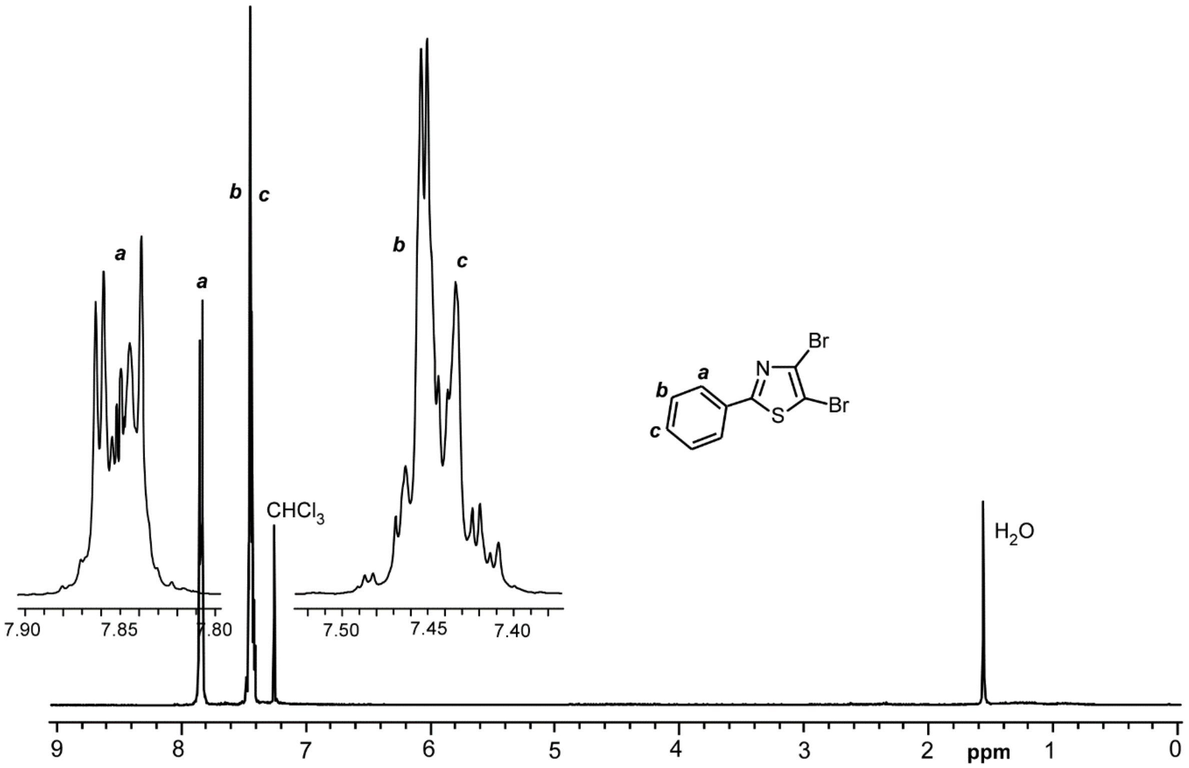

Figure S3. ^{1}H NMR Spectrum of 4,5-dibromo-2-phenylthiazole (**13**).

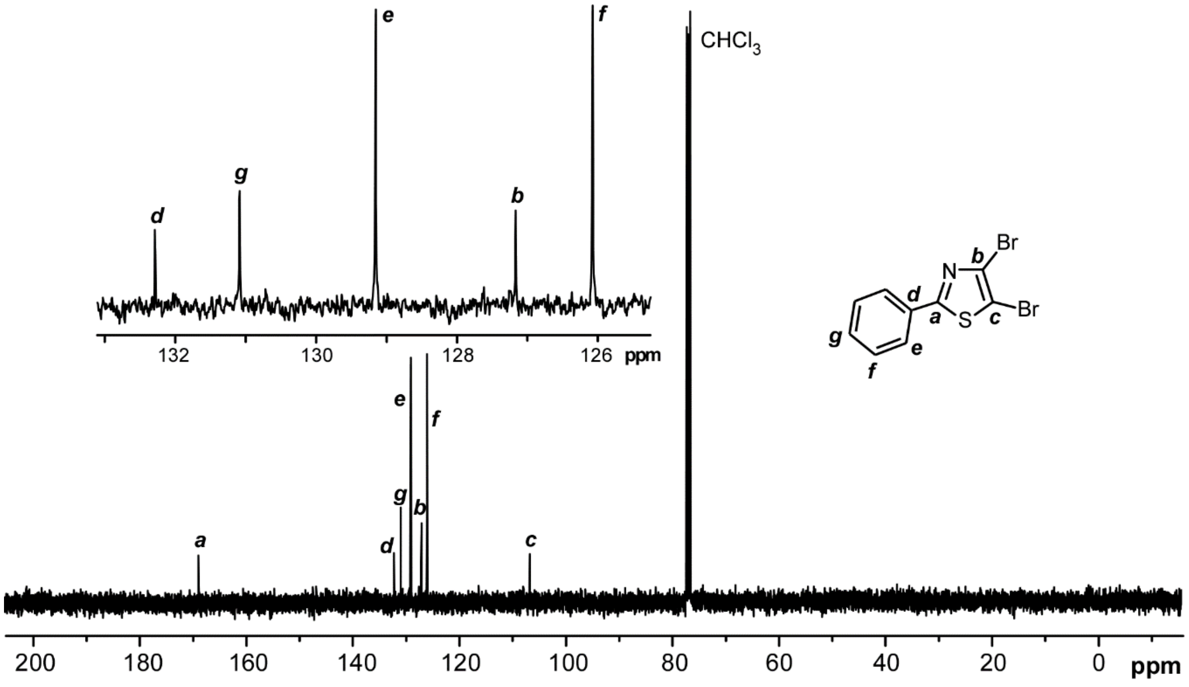

Figure S4. ^{13}C NMR Spectrum of 4,5-dibromo-2-phenylthiazole (**13**).

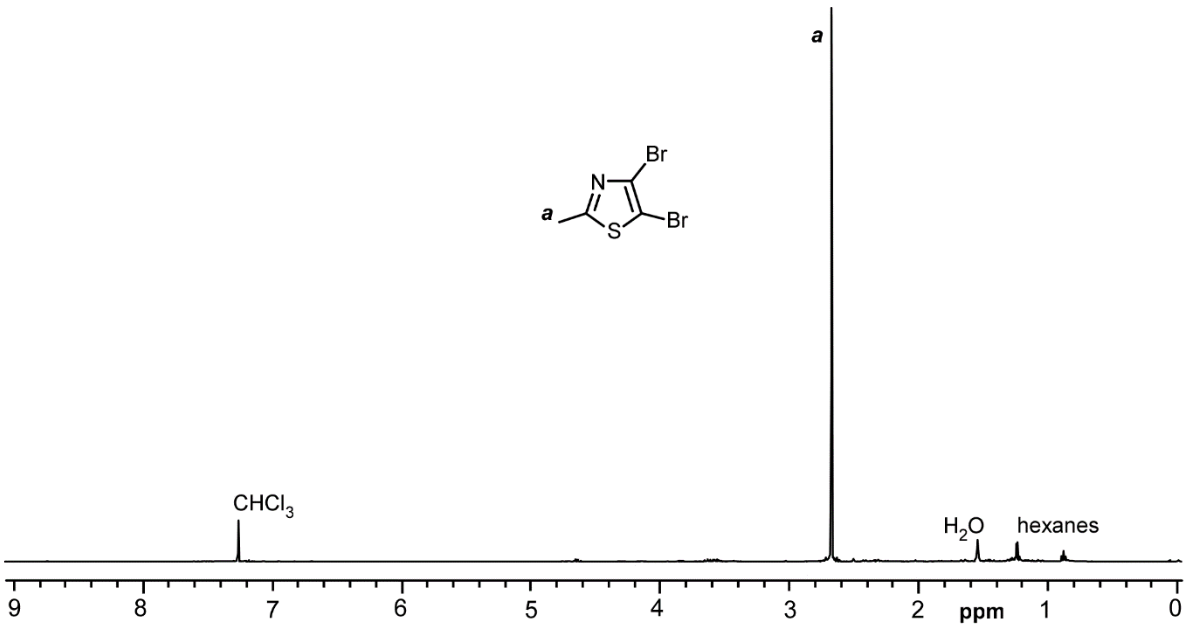

Figure S5. ^{1}H NMR Spectrum of 4,5-Dibromo-2-methylthiazole (**20**).

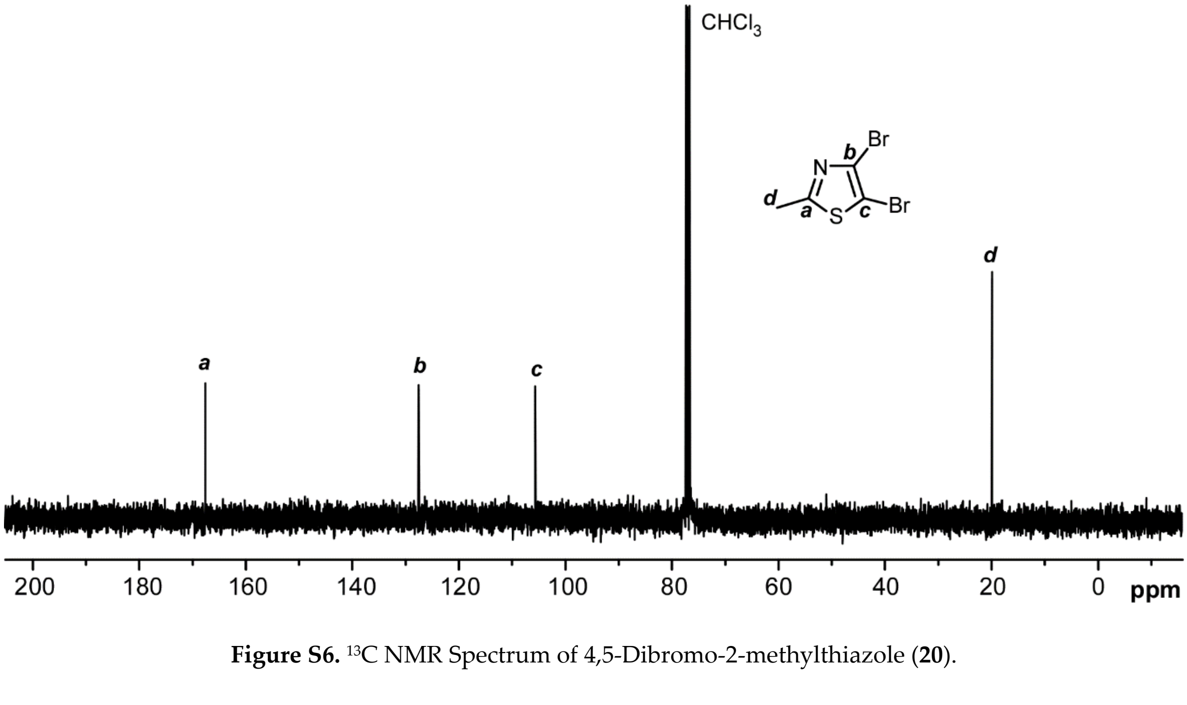

Figure S6. ^{13}C NMR Spectrum of 4,5-Dibromo-2-methylthiazole (**20**).

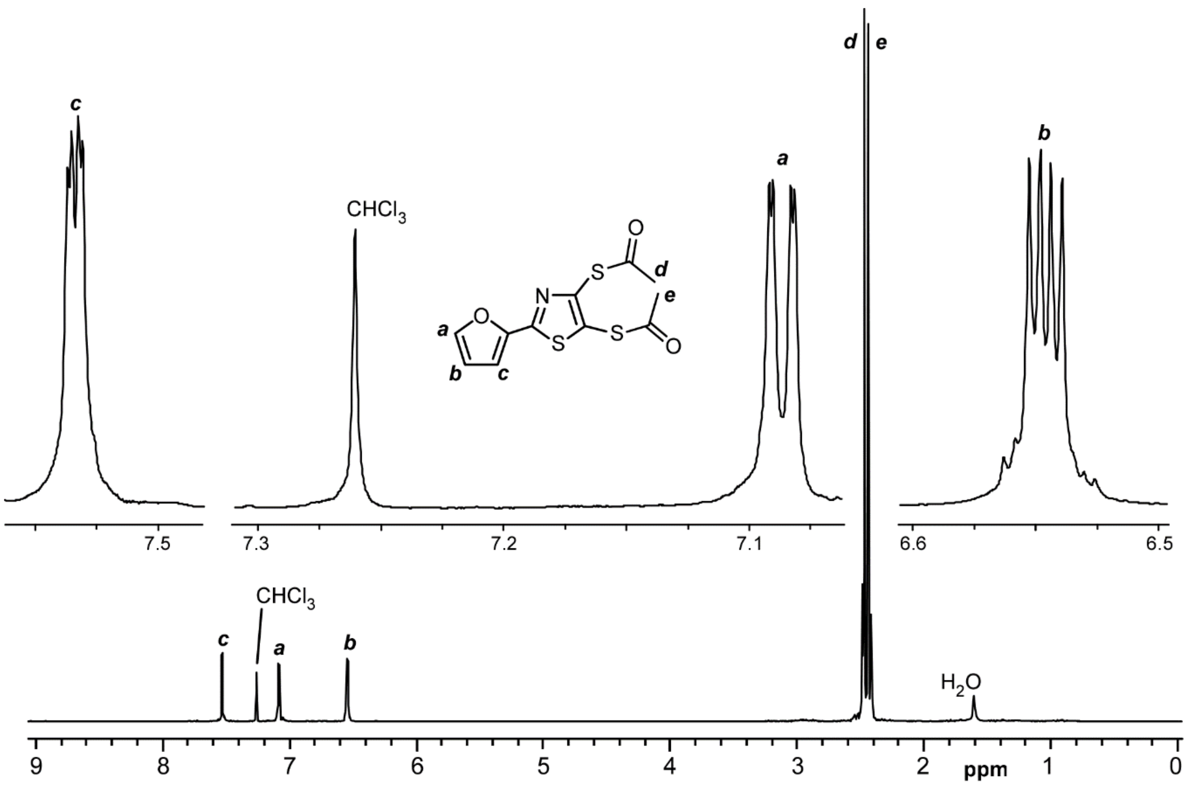

Figure S7. ^{1}H NMR Spectrum of 4,5-bis(thioacetate)-2-(2-furyl)thiazole (**15**).

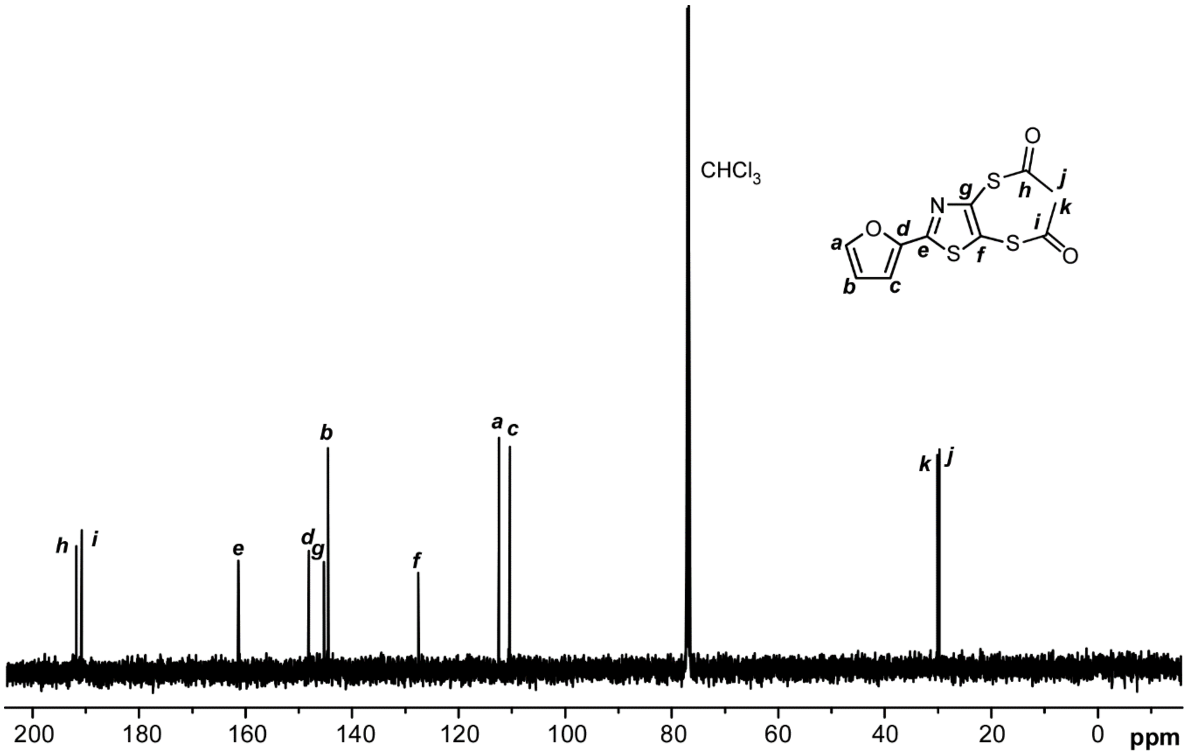

Figure S8. ^{13}C NMR Spectrum of 4,5-bis(thioacetate)-2-(2-furyl)thiazole (**15**).

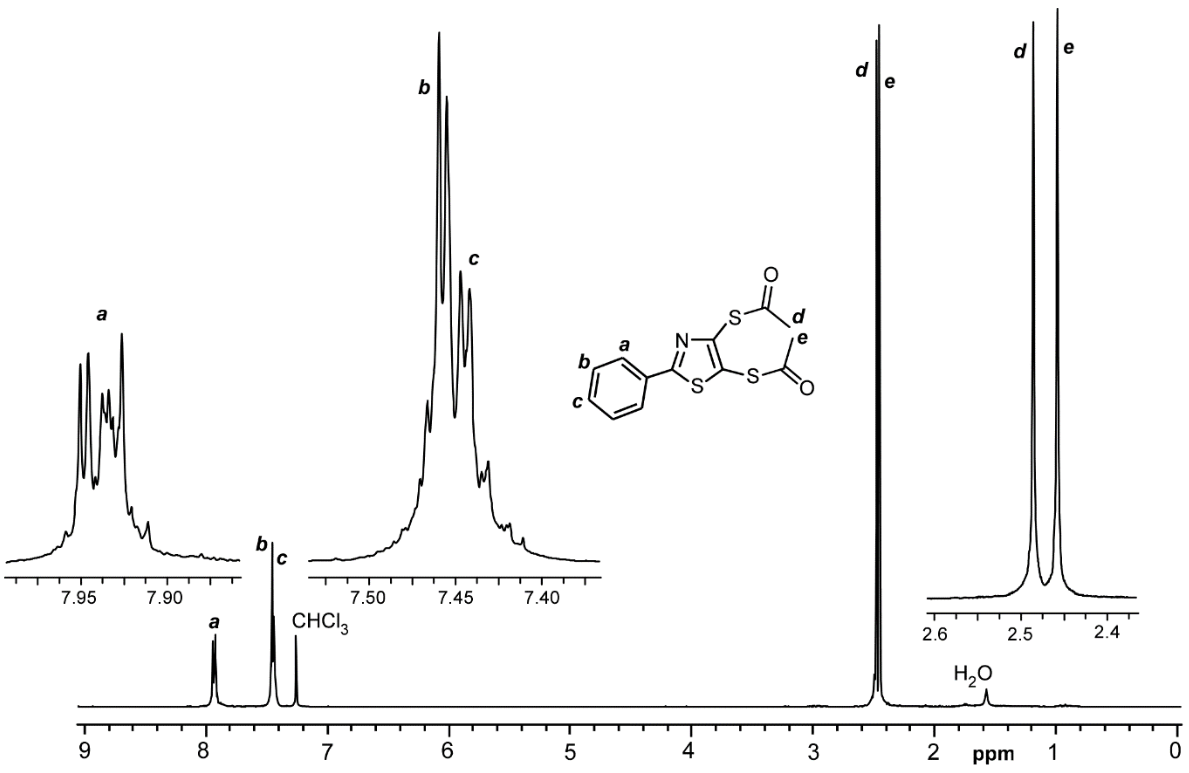

Figure S9. ^{1}H NMR Spectrum of 4,5-bis(thioacetate)-2-phenylthiazole (**16**).

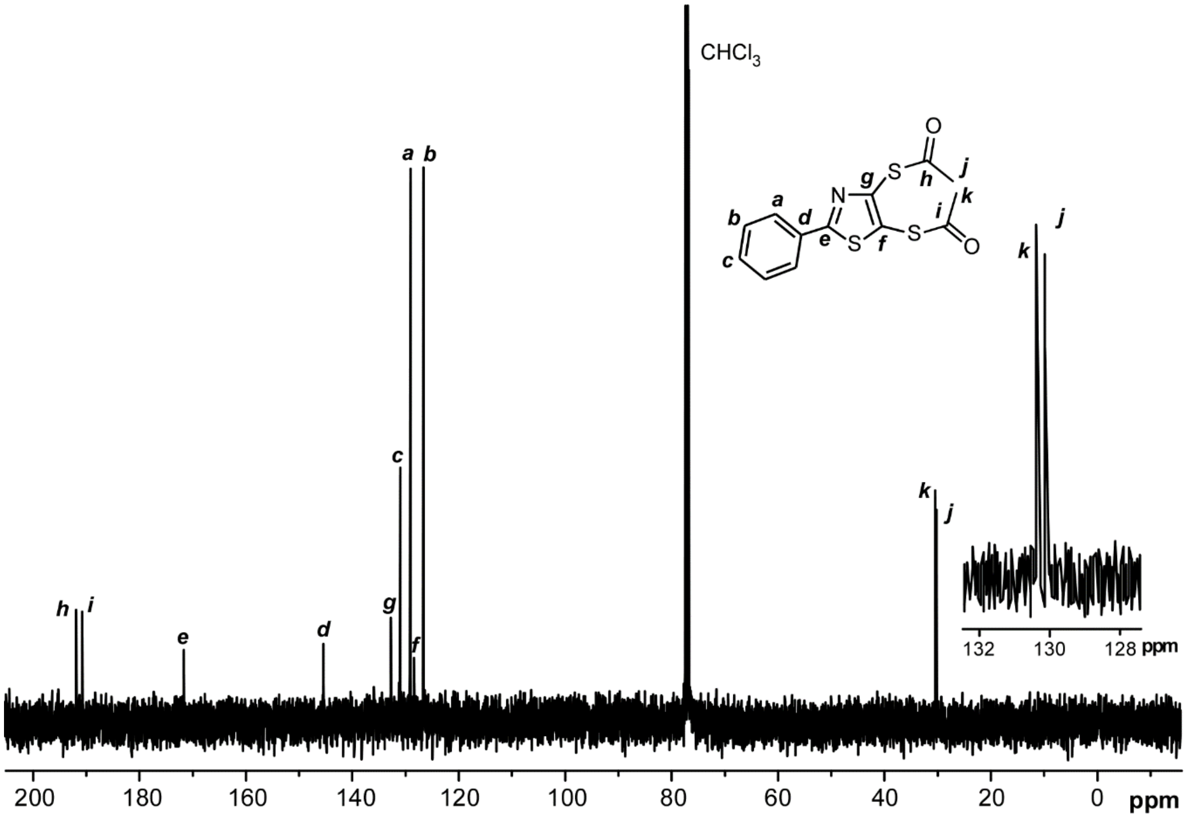

Figure S10. ^{13}C NMR Spectrum of 4,5-bis(thioacetate)-2-phenylthiazole (**16**).

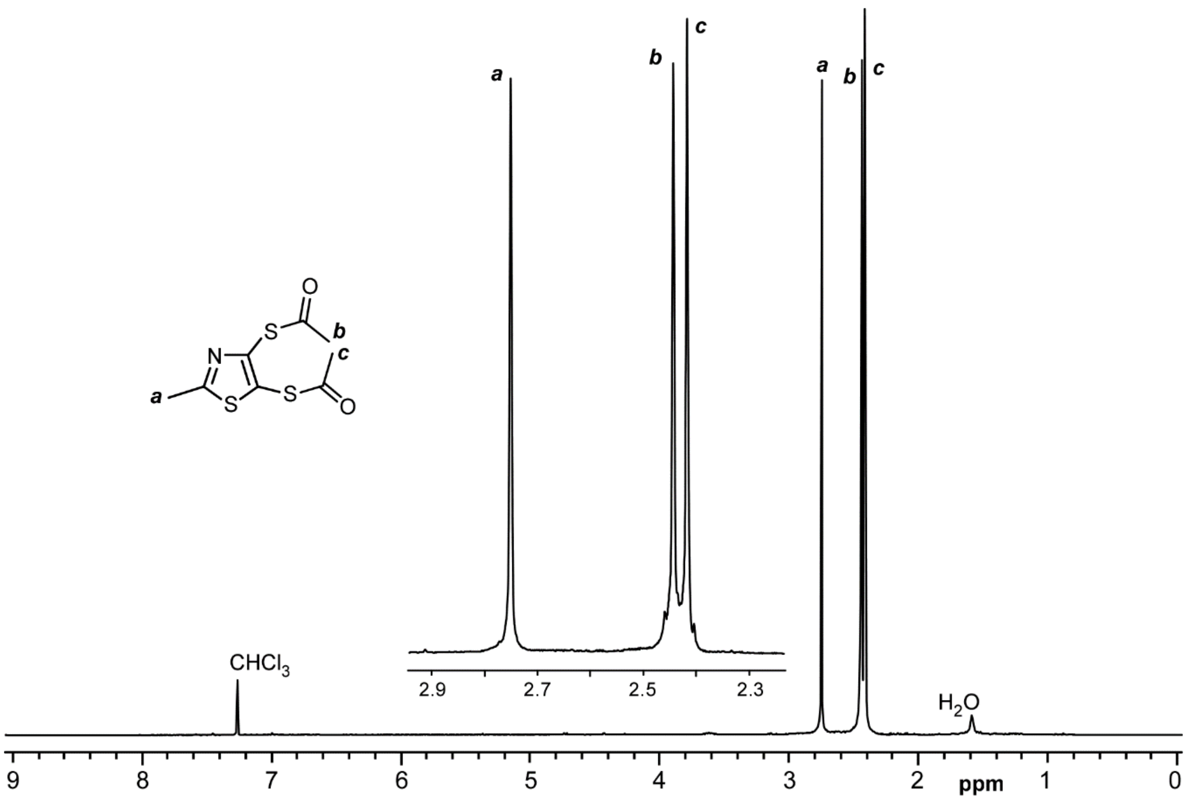

Figure S11. ^{1}H NMR Spectrum of 4,5-bis(thioacetate)-2-methylthiazole (**21**).

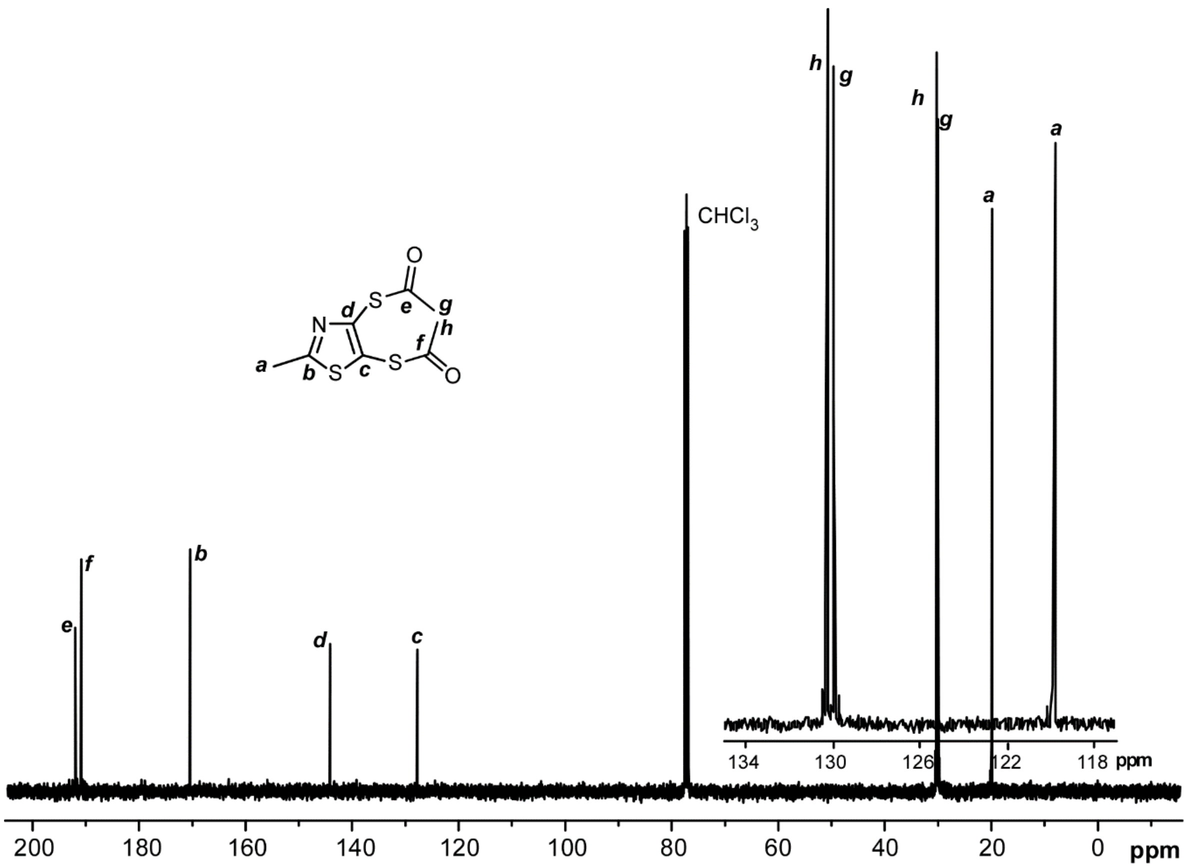

Figure S12. ^{13}C NMR Spectrum of 4,5-bis(thioacetate)-2-methylthiazole (**21**).

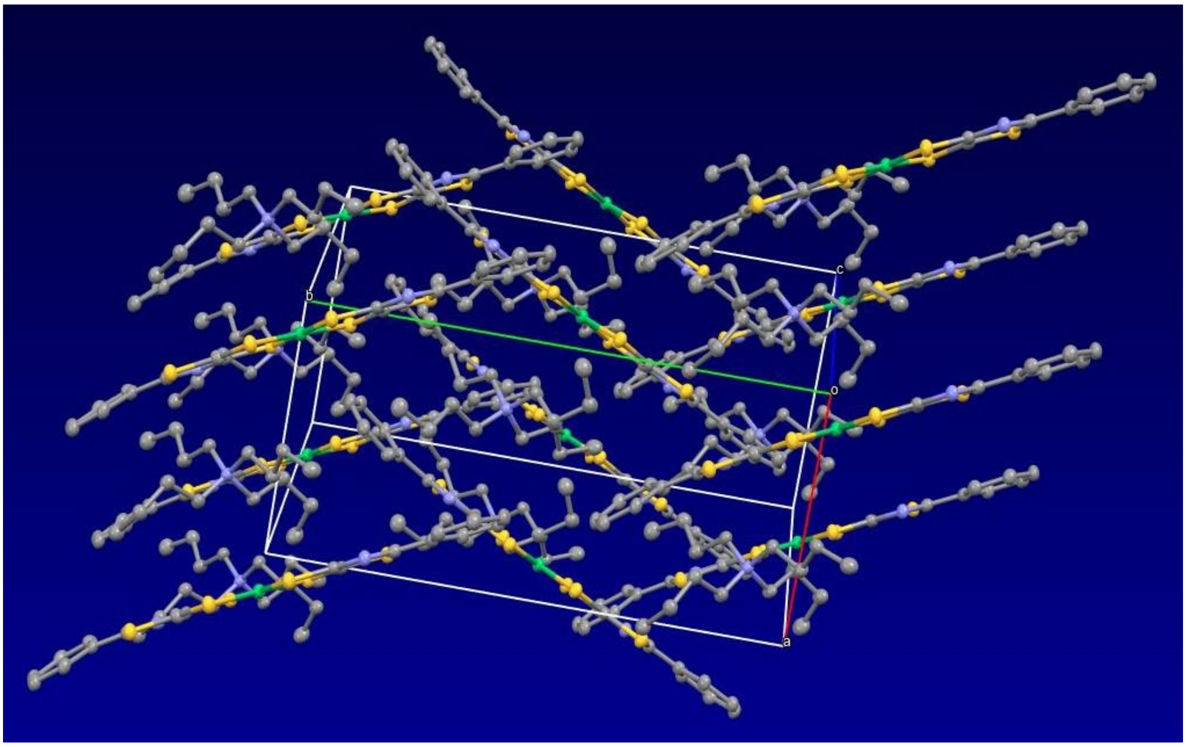

Figure S13. Crystal packing of [Bu_4N][**8**].

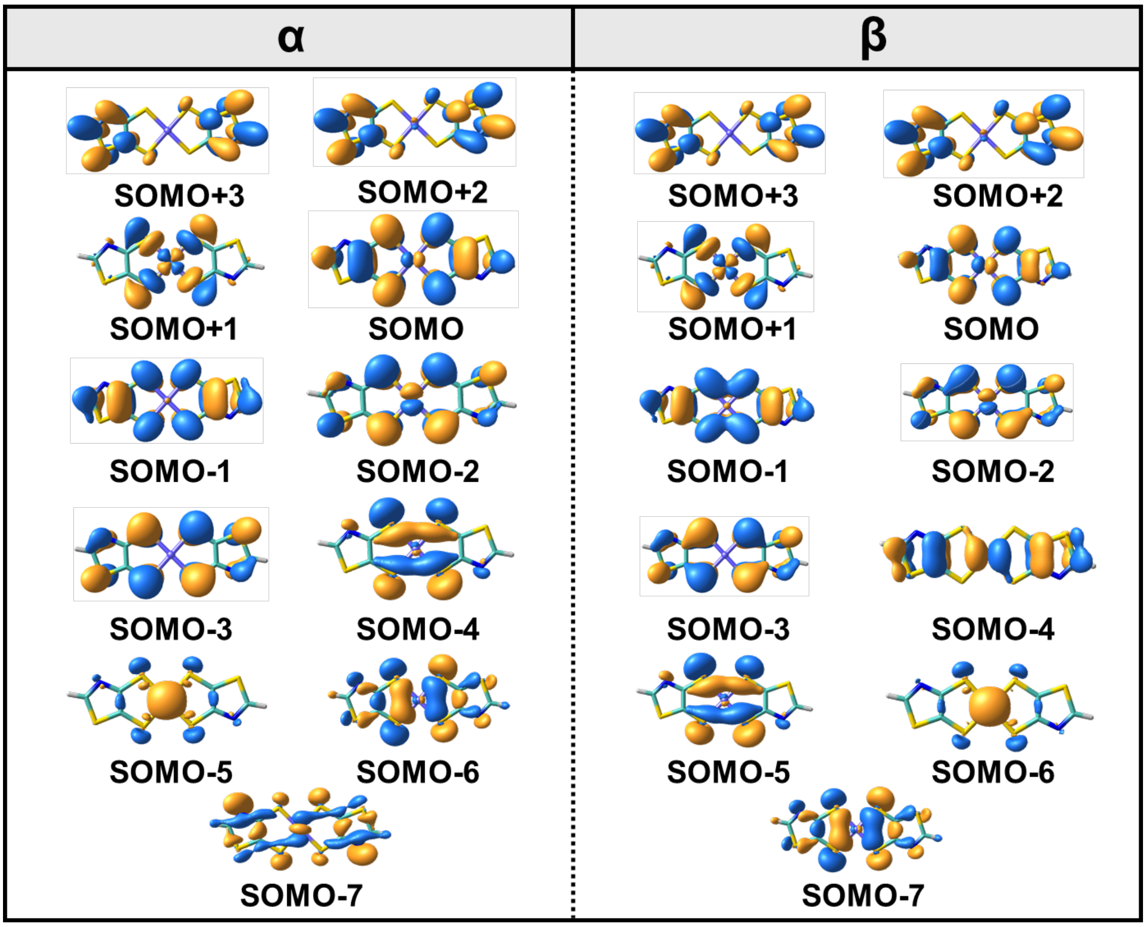

Figure S14. Electronic density contours of the MOs of the core nickel thiazoledithiolene (NiTzDT), calculated at the CAM-B3LYP-D3/def2-TZVP level of theory.

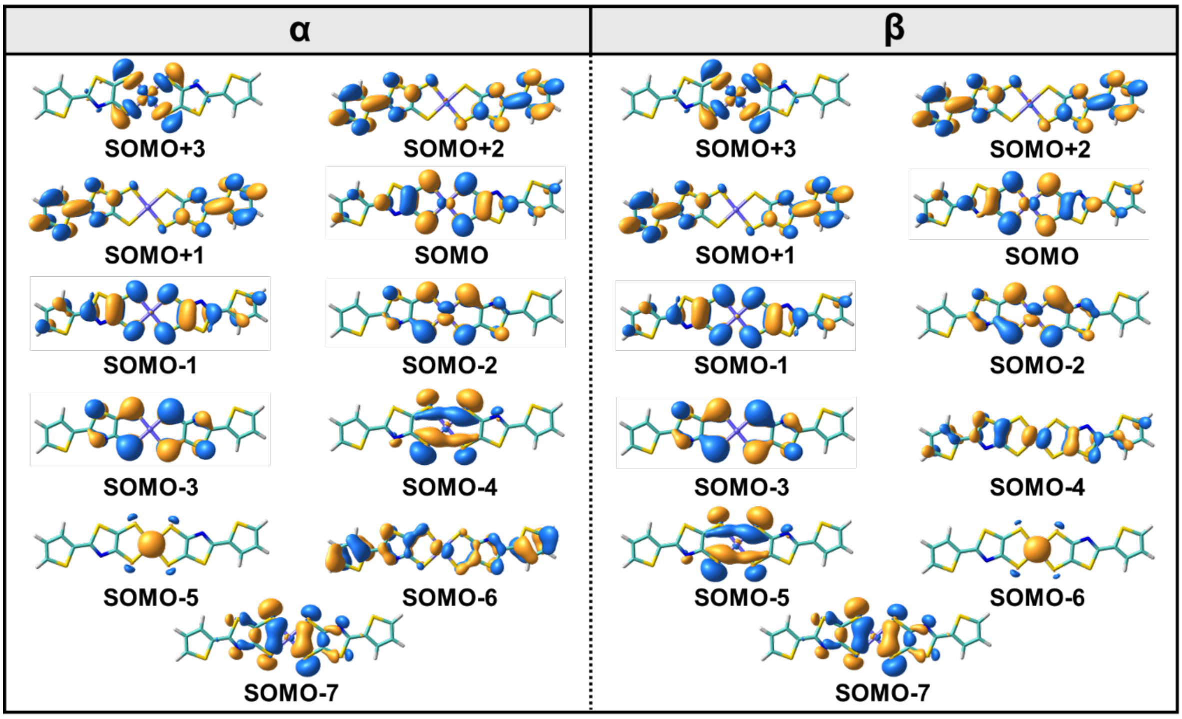

Figure S15. Electronic density contours of the MOs of the thiophene-extended complex **6**, calculated at the CAM-B3LYP-D3/def2-TZVP level of theory.

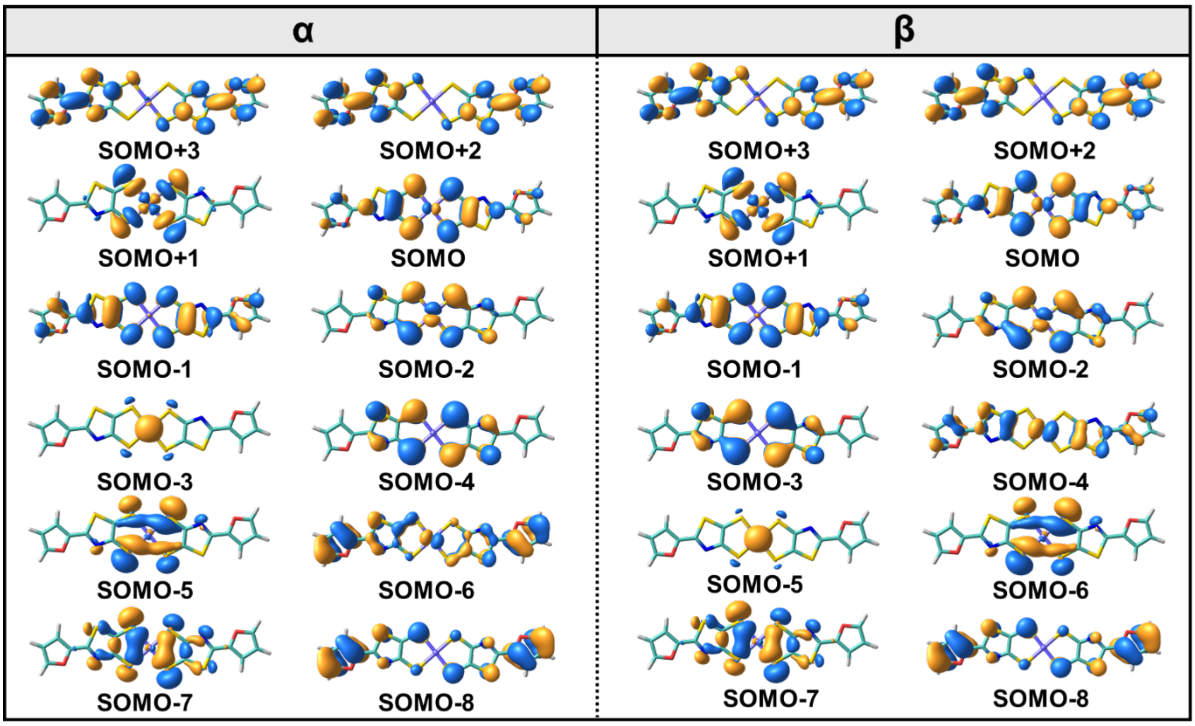

Figure S16. Electronic density contours of the MOs of the furan-extended complex **7**, calculated at the CAM-B3LYP-D3/def2-TZVP level of theory.

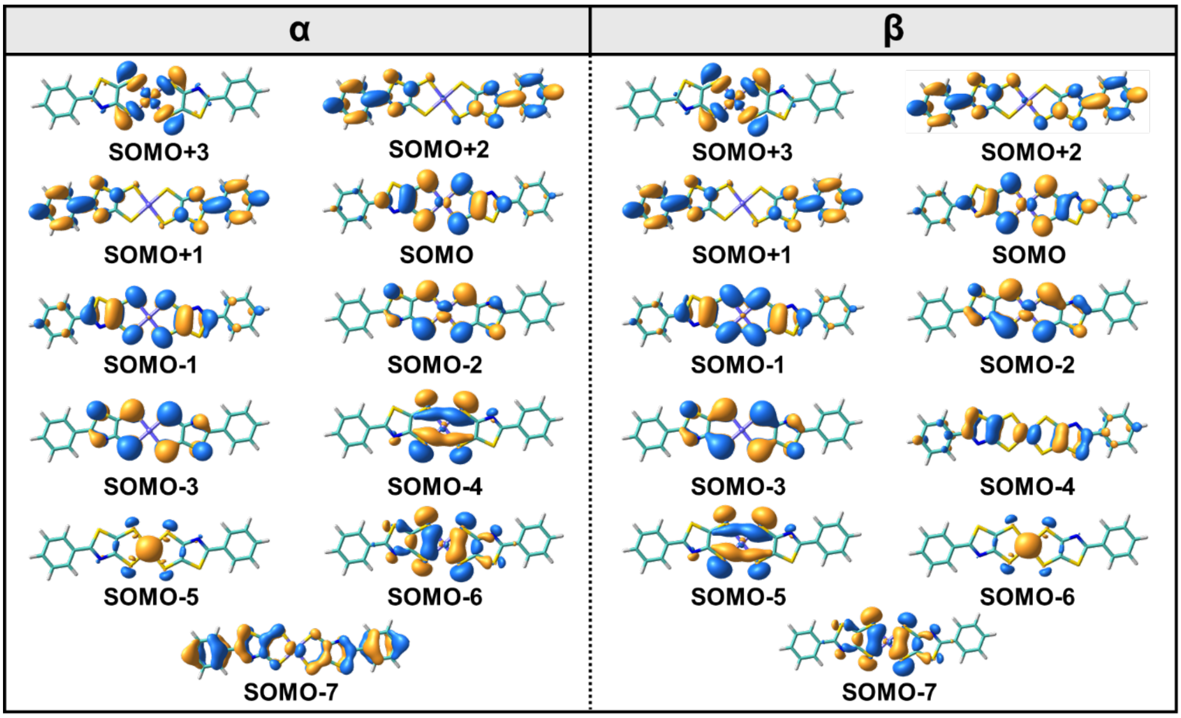

Figure S17. Electronic density contours of the MOs of the phenyl-extended complex **8**, calculated at the CAM-B3LYP-D3/def2-TZVP level of theory.

Table S1. TDDFT-calculated vertical excitation energies at UB3LYP-D3/def2-TZVP level of theory.

Compound	λ_{max}^{calc} (nm)	f_{calc}	Primary description	Transition
NiTzDT	947	0.13	SOMO-1β → SOMOβ (97%)	IVCT
	624	0.02	SOMO-3β → SOMOβ (96%)	LMCT
	461	0.01	SOMOα → SOMO+3α (58%)<br>SOMO-1α → SOMO+2α (13%)<br>SOMO-1β → SOMO+2β (21%)	MLCT<br>π → π*<br>π → π*
	356	0.04	SOMOα → SOMO+3α (35%)<br>SOMO-1β → SOMO+2β (48%)	MLCT<br>π → π*
	285	0.51	SOMO-5α → SOMO+1α (22%)<br>SOMO-2α → SOMO+3α (19%)<br>SOMO-6β → SOMO+1β (28%)<br>SOMO-2β → SOMO+3β (16%)	π → π*<br>MLCT<br>π → π*<br>MLCT
**6**	1066	0.28	SOMO-1β → SOMOβ (98%)	IVCT
	708	0.13	SOMOα → SOMO+1α (65%)	MLCT
	515	0.18	SOMOα → SOMO+1α (33%)<br>SOMO-1β → SOMO+2β (45%)	MLCT<br>π → π*
	443	0.16	SOMO-1α → SOMO+2α (46%)<br>SOMO-1β → SOMO+2β (33%)	π → π*<br>π → π*
	416	0.16	SOMO-1α → SOMO+2α (18%)<br>SOMO-2α → SOMO+1α (14%)<br>SOMO-2β → SOMO+1β (44%)	π → π*<br>MLCT<br>MLCT
	363	0.17	SOMO-8β → SOMOβ (43%)<br>SOMO-4β → SOMO+1β (13%)	LMCT<br>MLCT

Table S1 (continued).

Compound	λ_{max}^{calc} (nm)	f_{calc}	Primary description	Transition
**8**	1043	0.28	SOMO-1β → SOMOβ (98%)	IVCT
	662	0.14	SOMOα → SOMO+2α (62%)	MLCT
	489	0.20	SOMOα → SOMO+2α (29%)<br>SOMO-1β → SOMO+2β (50%)	MLCT<br>π → π*
	405	0.18	SOMO-1α → SOMO+3α (21%)<br>SOMO-2β → SOMO+1β (42%)	MLCT<br>π → π*
	353	0.24	SOMO-2α → SOMO+2α (24%)<br>SOMO-10β → SOMOβ (45%)	π → π*<br>LMCT
	320	0.23	SOMO-3α → SOMO+3α (68%)	π → π*
**7**	1052	0.26	SOMO-1β → SOMOβ (98%)	IVCT
	662	0.11	SOMOα → SOMO+2α (59%)	MLCT
	477	0.18	SOMOα → SOMO+2α (39%)<br>SOMO-1β → SOMO+3β (43%)	MLCT<br>π → π*
	405	0.18	SOMO-1α → SOMO+3α (29%)<br>SOMO-2α → SOMO+2α (14%)<br>SOMO-2β → SOMO+2β (29%)	π → π*<br>MLCT<br>MLCT
	356	0.17	SOMO-2α → SOMO+2α (46%)<br>SOMO-2β → SOMO+2β (23%)	MLCT<br>MLCT
	313	0.19	SOMO-4α → SOMO+3α (57%)<br>SOMO-3β → SOMO+3β (12%)	π → π*<br>π → π*

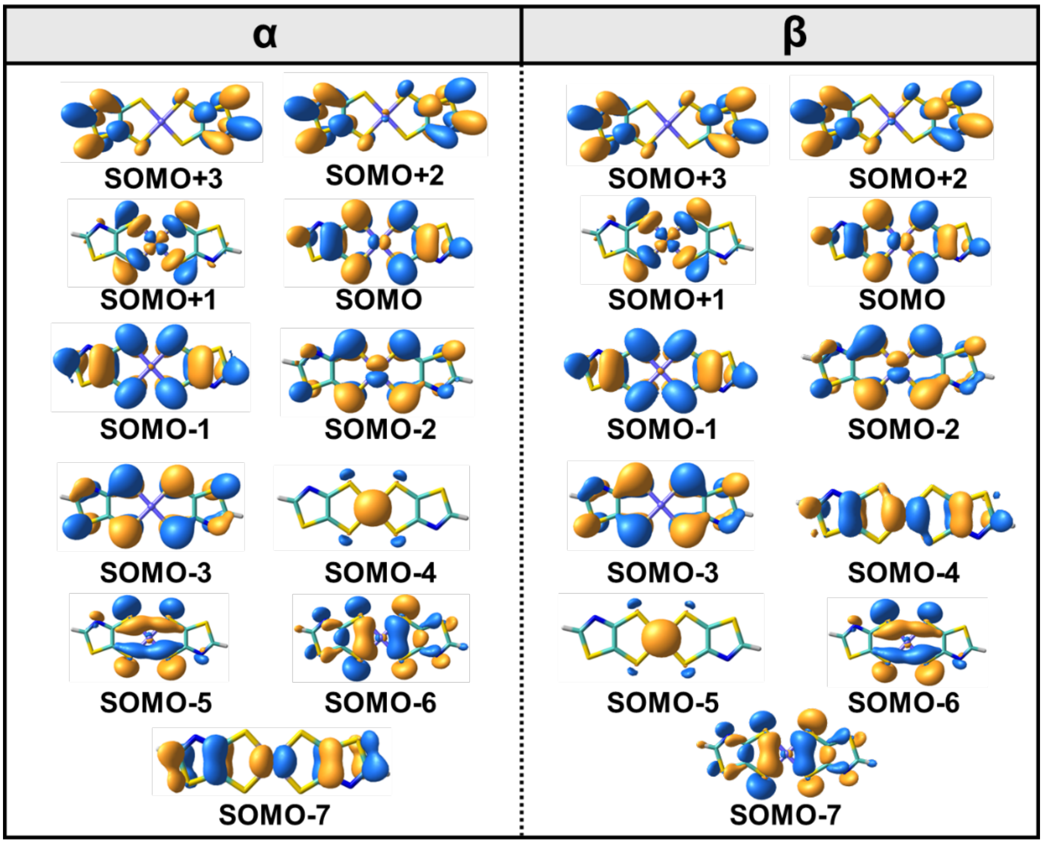

Figure S18. Electronic density contours of the MOs of the core nickel thiazoledithiolene (NiTzDT), calculated at the UB3LYP-D3/def2-TZVP level of theory.

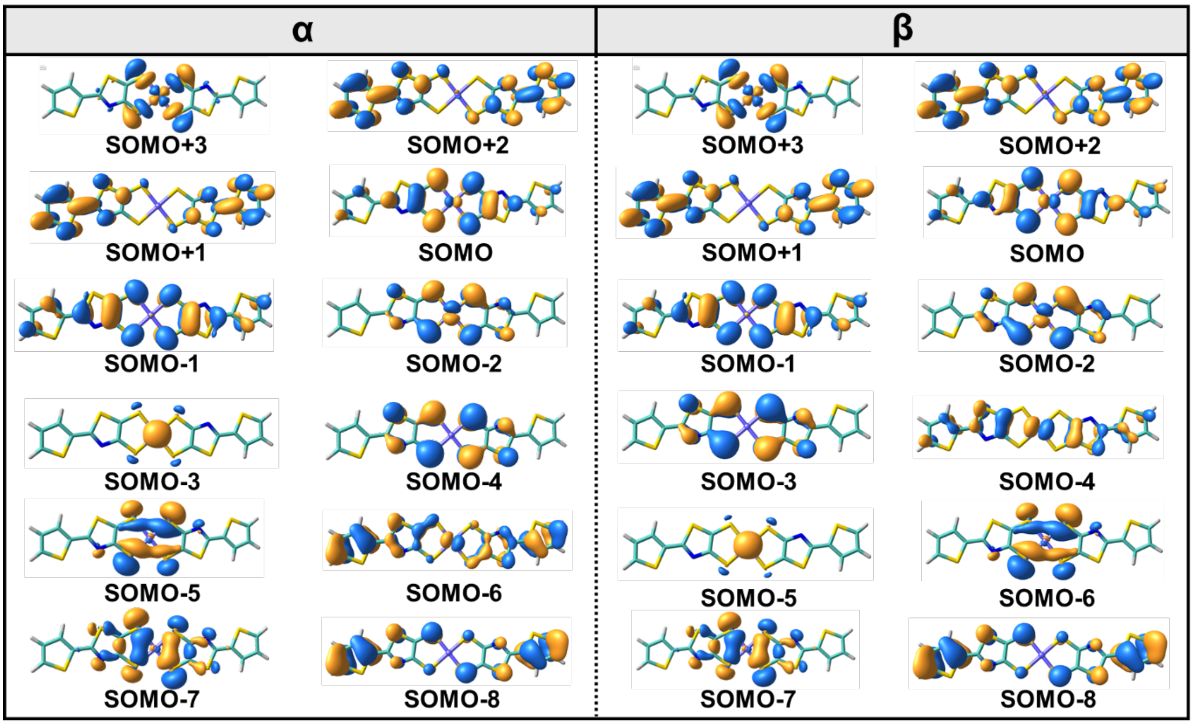

Figure S19. Electronic density contours of the MOs of the thiophene-extended complex **6**, calculated at the UB3LYP-D3/def2-TZVP level of theory.

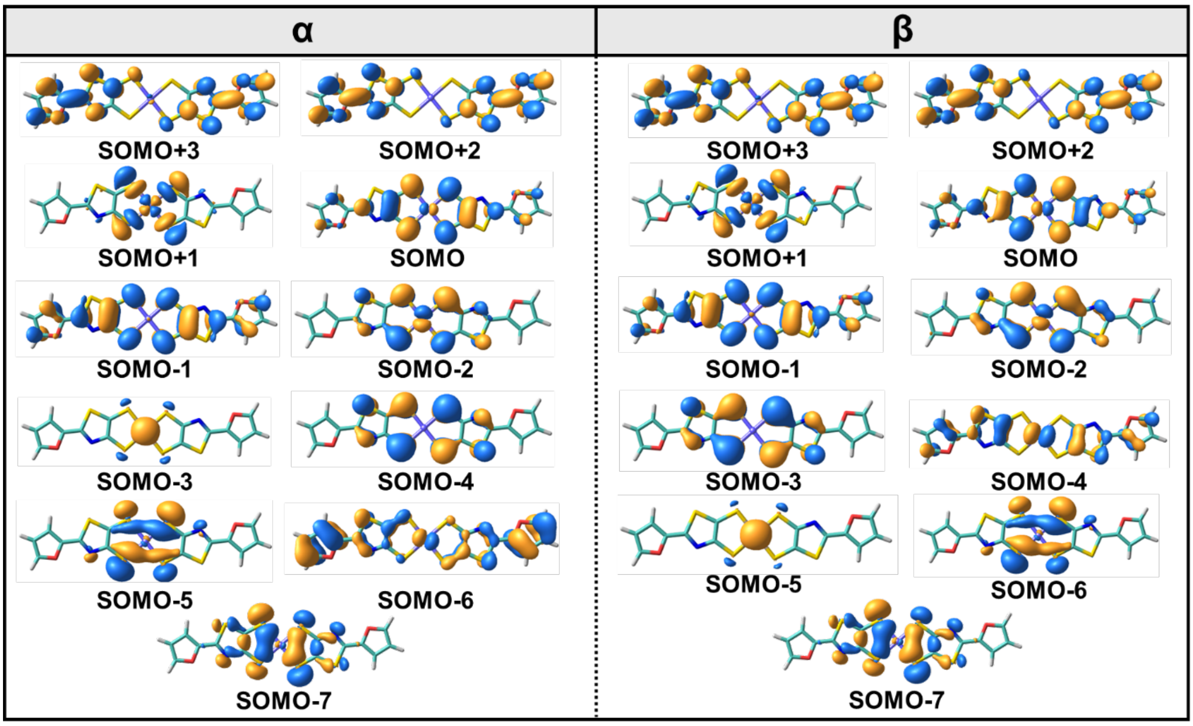

Figure S20. Electronic density contours of the MOs of the furan-extended complex **7**, calculated at the UB3LYP-D3/def2-TZVP level of theory.

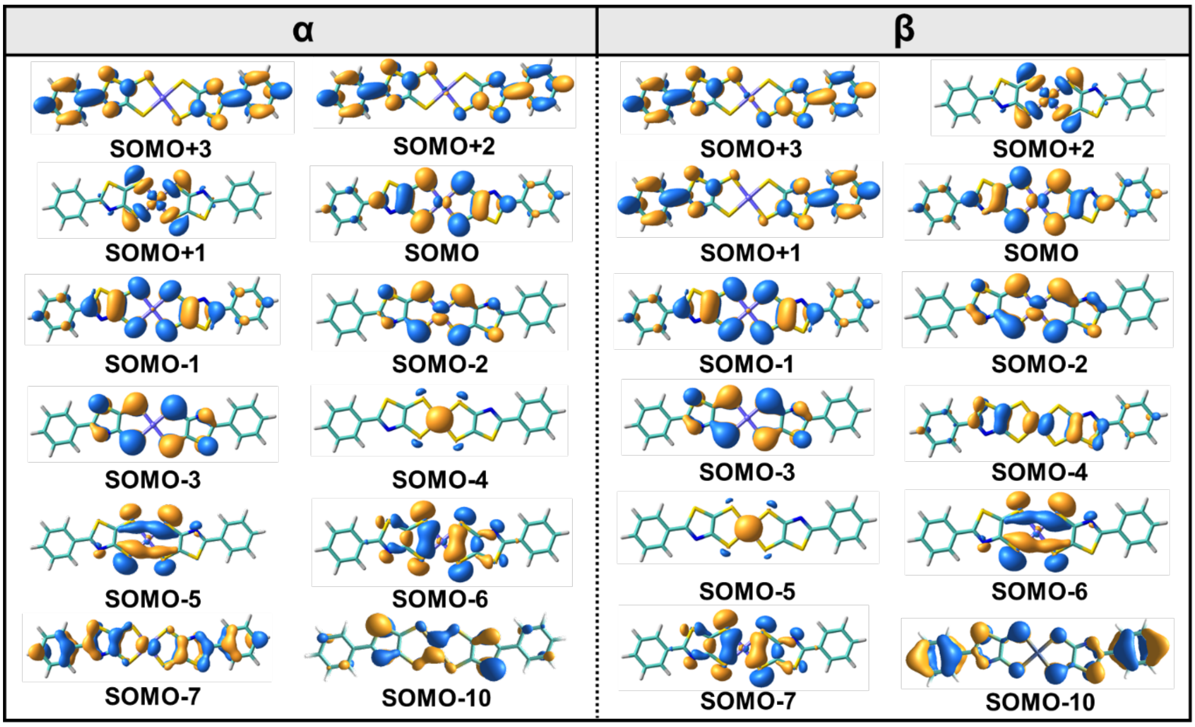

Figure S21. Electronic density contours of the MOs of the phenyl-extended complex **8**, calculated at the UB3LYP-D3/def2-TZVP level of theory.
